# Evaluation of Organo [^18^F]Fluorosilicon Tetrazine as a Prosthetic Group for the Synthesis of PET Radiotracers

**DOI:** 10.3390/molecules25051208

**Published:** 2020-03-07

**Authors:** Sofia Otaru, Surachet Imlimthan, Mirkka Sarparanta, Kerttuli Helariutta, Kristiina Wähälä, Anu J. Airaksinen

**Affiliations:** 1 Department of Chemistry, Radiochemistry, University of Helsinki, 00100 Helsinki, Finland; 2 Department of Biochemistry and Developmental Biology, Faculty of Medicine, University of Helsinki, 00100 Helsinki, Finland; 3 Turku PET Centre, Department of Chemistry, University of Turku, 20500 Turku, Finland

**Keywords:** fluorine-18, positron emission tomography (PET), defluorination, isotopic exchange, silicon-based fluoride acceptor, bioorthogonal chemistry, tetrazine, inverse electron-demand Diels-Alder ligation

## Abstract

Fluorine-18 is the most widely used positron emission tomography (PET) radionuclide currently in clinical application, due to its optimal nuclear properties. The synthesis of ^18^F-labeled radiotracers often requires harsh reaction conditions, limiting the use of sensitive bio- and macromolecules as precursors for direct radiolabeling with fluorine-18. We aimed to develop a milder and efficient in vitro and in vivo labeling method for trans-cyclooctene (TCO) functionalized proteins, through the bioorthogonal inverse-electron demand Diels-Alder (IEDDA) reaction with fluorine-18 radiolabeled tetrazine ([^18^F]SiFA-Tz). Here, we used TCO-modified bovine serum albumin (BSA) as the model protein, and isotopic exchange (IE) (^19^F/^18^F) chemistry as the labeling strategy. The radiolabeling of albumin-TCO with [^18^F]SiFA-Tz ([^18^F]**6**), providing [^18^F]fluoroalbumin ([^18^F]**10**) in high radiochemical yield (99.1 ± 0.2%, *n* = 3) and a molar activity (MA) of 1.1 GBq/µmol, confirmed the applicability of [^18^F]**6** as a quick in vitro fluorination reagent for the TCO functionalized proteins. While the biological evaluation of [^18^F]**6** demonstrated defluorination in vivo, limiting the utility for pretargeted applications, the in vivo stability of the radiotracer was dramatically improved when [^18^F]**6** was used for the radiolabeling of albumin-TCO ([^18^F]**10**) in vitro, prior to administration. Due to the detected defluorination in vivo, structural optimization of the prosthetic group for improved stability is needed before further biological studies and application of pretargeted PET imaging.

## 1. Introduction 

Fluorine-18 is an ideal radionuclide for labeling of radiopharmaceuticals for positron emission tomography (PET), due to its nuclear and physical characteristics, including the relatively long half-life (109.7 min), the low energy levels of emitted positrons (*E*max = 0.635 MeV), and high positron decay probability (97%) [1]. Fluorine-18 can be produced via the ^18^O(p,n)^18^F reaction, by irradiating ^18^O-enriched water with protons, yielding high molar activity [^18^F]fluoride, in aqueous solutions. Several advances in ^18^F-fluorinations, such as metal-mediated (e.g., Pd, Cu, Ag) aromatic, aliphatic, and aryl boronic ester radiofluorinations, as well as TiO_2_-catalyzed ^18^F-fluorinations in aqueous media, have been recently presented [2,3,4]. The incorporation of nucleophilic [^18^F]fluoride into molecules generally requires alkaline conditions and elevated temperatures. Faster, milder, and more selective radiolabeling methodologies are desired, especially for the radiolabeling of compounds sensitive to temperature and higher pH. Fast and efficient catalyst-free click-reactions, such as the inverse electron-demand Diels-Alder (IEDDA) reaction, have been applied as effective tools for the selective incorporation of radiolabels, such as ^18^F, into bio- and macromolecules, via small radiolabeled prosthetic groups. Other widely known click-reactions, such as the copper(I)-catalyzed azide-alkyne cycloaddition (CuAAC) and the stainpromoted azide-alkyne cycloaddition (SPAAC) have been the starting point for the modification of chemoselective biomolecules [5]. The CuAAC reaction was first reported in 2002 by Sharpless et al. [6] and Meldal et al. [7]. The aim of these studies was to utilize the CuAAC reaction for the formation of an enormous variety of five-membered heterocycles, triazoles, and peptide derivatives. The use of CuAAC led to the investigation on the utility of these highly efficient reactions for the labeling of biomolecules in living systems. However, the toxicity of copper limited the feasibility of CuAAC in biological applications. SPAAC was developed in 2004 by Bertozzi et al. who demonstrated the chemical modification of live Jurkat cells with an azide-modified sugar for the subsequent conjugation of alkyne-biotin for fluorescent labeling with FITC-avidin, without any apparent decline in cell viability [8]. Since then, SPAAC has served as a catalyst-free alternative to overcome the cytotoxicity concerns of the CuAAC reaction. However, both CuAAC and SPAAC have relatively slow reaction kinetics, which renders the reactions unsuitable for labeling of biomolecules in a living system, for applications lile in vivo pretargeting. In 1959, Lindsey et al. reported the ability of tetrazines to react chemoselectively with unsaturated compounds through a 1,4-cycloaddition reaction [9]. These findings introduced a new and highly reactive bioorthogonal IEDDA click-reaction as a pivotal tool for synthetic modification of biomolecules. 

The bioorthogonal IEDDA-reaction has been successfully used for various pretargeted in vivo radiolabeling applications [10,11,12]. Pretargeted imaging has found exceptional utility with imaging agents with slow pharmacokinetics, such as antibodies and nanomaterials, which when directly radiolabeled would require the use of long-lived radioisotopes (such as ^89^Zr or ^111^In, both with ~3-day physical half-lives), to track their biodistribution in vivo. In the pretargeted approach, the targeting vector (such as an IgG antibody) is first modified by one reactant of the IEDDA reaction, and allowed to distribute in the body after administration. Next, it is tracked using the other reactant that is radiolabeled with a short-lived radioisotope, with improved image contrast and lower radiation burden to the subject [13,14]. The fastest IEDDA-reaction reported so far is from the conjugation between tetrazine (Tz) and trans-cyclooctene (TCO) (*k* ≈ 10^6^ M^−1^ s^−1^) [5], rendering this reactive pair of utmost interest in the fields of chemical biology, nuclear imaging, and radiotracer development. However, the sensitivity of tetrazines towards alkaline conditions and elevated temperature renders the direct radiolabeling of tetrazines with fluorine-18 challenging. Therefore, the use of prosthetic groups for radiolabeling, such as the glycoconjugate [^18^F]-5-fluoro-5-deoxyribose (FDR) or Al[^18^F]F [15,16,17], is necessary to ensure that radiolabeling conditions preserve the reactivity of the Tz. 

The silicon-fluoride acceptor (SiFA) chemistry relies on ^19^F/^18^F-isotopic exchange for introducing fluorine-18 into radiotracers, and has emerged as a fast and mild radiolabeling tool, especially for sensitive molecules [18]. The small lipophilic SiFA compounds mainly utilized in the radiolabeling of larger constructs such as peptides, proteins, and nanoparticles, in most cases have demonstrated excellent stability against in vivo defluorination [19,20,21,22,23]. The lipophilic character of the SiFA-derivatives can be used to tailor the pharmacokinetics of biomolecular tracers. To our knowledge, there is only one compound containing SiFA bound to a tetrazine, SiFA-OTz, which has been reported to date, but its enzymatic stability in vitro and in vivo has not yet been studied [24]. Nevertheless, the tracer SiFA-OTz demonstrated good stability under the radiolabeling conditions. We have reported the development of highly stable and highly hydrophilic ^18^F-tetrazines from sugar analogues, such as [^18^F]fluorodeoxyribose ([^18^F]FDR-Tz) and 2-deoxy-2-[^18^F]fluoro-*D*-glucose ([^18^F]FDG-Tz) [17,25]. We also previously studied the feasibility of utilizing the ^18^F-FDR-Tz for the in vivo IEDDA pretargeting of antibodies and nanoparticles and were able to prove this approach to be highly successful [26,27].

Here, we investigated the use of a SiFA as a reaction strategy for the [^18^F]fluorination of tetrazines, under mild reaction conditions, with the possibility to yield a more hydrophobic tetrazine variant for the regulation of the pharmacokinetics of biomolecules labeled with [^18^F]SiFA-Tz. The aim of this study was to investigate [^18^F]SiFA-Tz as a standalone tracer, to reveal its potential for pretargeted imaging and its applicability for the rapid in vitro radiolabeling of TCO-containing biomolecules, under physiological conditions.

## 2. Results 

### 2.1. Chemistry

**Scheme 1:**
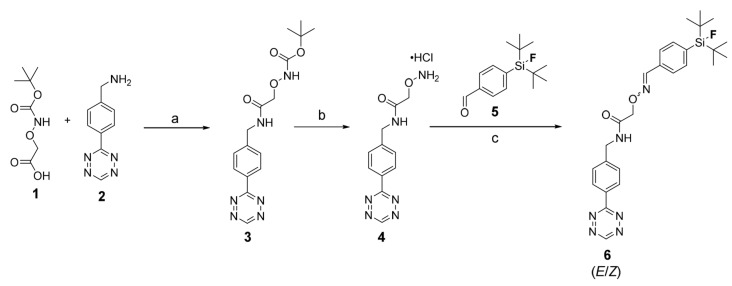
Synthesis of the precursor SiFA-Tz (**6**). Reagents and conditions: (**a**) HATU, DIPEA, DMF, room temperature, 20 h, Argon (65%), (**b**) 1 M HCl Et_2_O 25 °C, 24 h, MeOH (65%), and (**c**) Aniliniumacetate-buffer pH 4.6, 25 °C, 15 min (90%). (HATU; 1-[Bis(dimethylamino)methylene]-1H-1,2,3-triazolo[4,5-b]pyridinium 3-oxid hexafluorophosphate, DIPEA; N,N-Diisopropylethylamine, DMF; Dimethylformamide).

SiFA-Tz (**6**) was synthesized via a three-step route, providing good yields for each step (Scheme 1) [14]. The first step comprised of an amide coupling reaction under argon, between a carboxylic acid and an amine, forming the t-Boc protected aminooxy-tetrazine **3**, in 65% yield after silica gel column chromatography purification. The next reaction step was the deprotection of **3** with hydrochloric acid in methanol, to give **4** as a pink solid in cold diethyl ether in 65% yield. This hydrochloric salt intermediate was used such for the next reaction, the oxime bond formation between compounds **4** and **5** generating an imine double bond such as *E*- and *Z*-isomers of SiFA-Tz (**6**) [28]. The reaction mixture was purified with semi-preparative HPLC, providing the desired product **6** in 90% yield. ^1^H-NMR, ^13^C-NMR, ^19^F-NMR spectra and ESI-TOF-MS were acquired for characterization of the final product, SiFA-Tz (**6**).

**Scheme 2:**
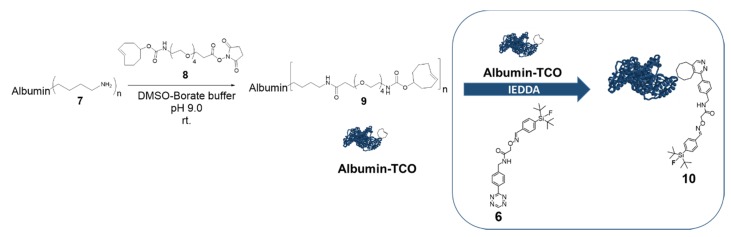
Functionalization of bovine serum albumin (**7**) with trans-cyclooctene (**8**) to form albumin- trans-cyclooctene (TCO) (**9**), followed by the inverse-electron demand Diels-Alder (IEDDA) cycloaddition with SiFA-Tz (**6**) to form fluoroalbumin (**10**) (molar ratio 1:1.5 SiFA-Tz:fluoroalbumin).

Bovine serum albumin (**7**) was functionalized with *N*-hydroxysuccinimide (NHS) ester of transcyclooctene (TCO) (200 eq), from the lysine residues available in the protein structure (Scheme 2). The analysis of the TCO:albumin ratio was carried out with MALDI-MS, which revealed a TCO:albumin ratio 40:1. The MALDI-MS analysis indicates that 65% of the total 62 lysine residues were functionalized with a TCO in one albumin molecule. The IEDDA cycloaddition product of SiFA-Tz (**6**) and albumin-TCO (**9**), fluoroalbumin (**10**), was synthesized as a reference for the radiolabeling studies by mixing the two reagents together at room temperature.

### 2.2. Radiochemistry

**Scheme 3:**
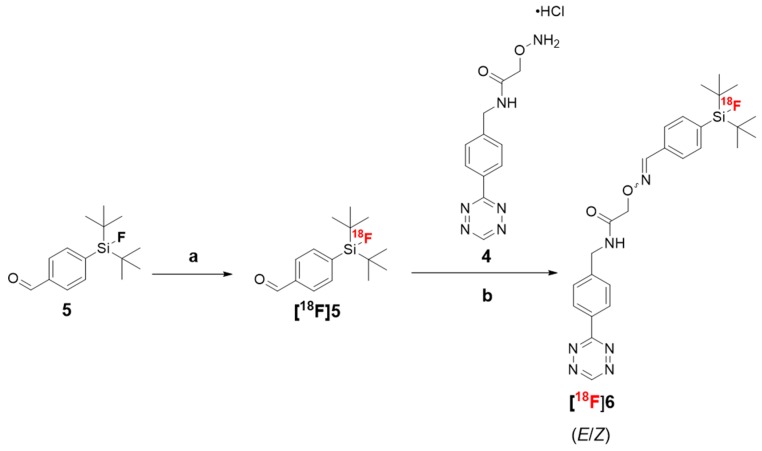
Radiosynthesis of [^18^F]SiFA-Tz ([^18^F]**6**) by a two-step method. Reagents and conditions: (**a**) ACN, K[^1 8^F]F-[K2.2.2], (RCY 99%) and (**b**) anilinium acetate buffer pH 4.6, 25 °C, 15 min (RCY 43%).

**Figure 1:**
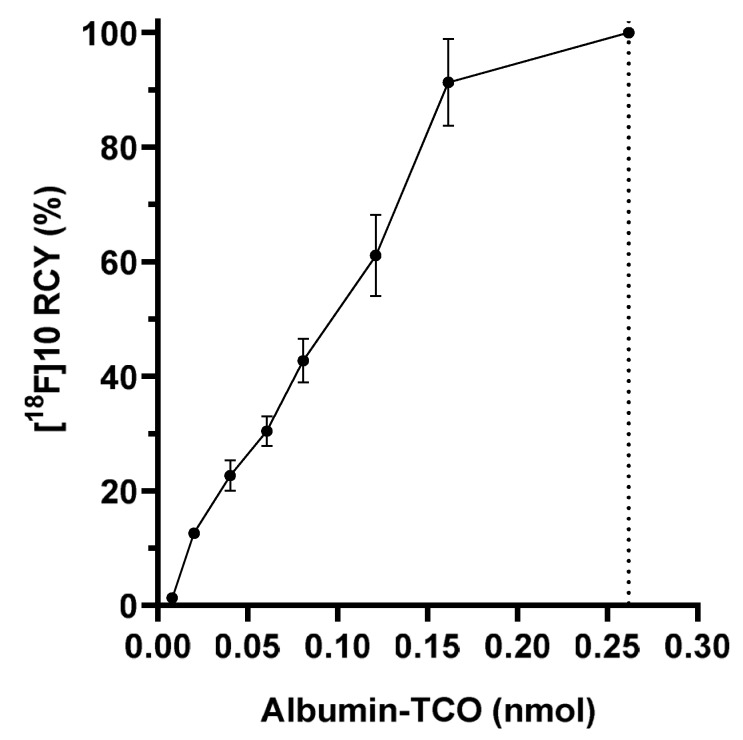
Radiochemical yield (RCY) of [^18^F]fluoroalbumin ([^18^F]**10**) during titration of [^18^F]SiFA-Tz ([^18^F]**6**), with albumin-TCO revealed a maximal radiochemical yield of >99% with 0.26 nmol of albumin-TCO.

Two different synthetic sequences were investigated for the radiosynthesis of [^18^F]**6**—a one–step direct radiolabeling of compound **6** and a two–step method where we first radiolabeled the SiFA moiety **5**, before linking it to the tetrazine **4** (Scheme 3). The one-step radiolabeling of **6**, resulted in 21% radiochemical yield (*n* = 1), but the yield was detected to decrease rapidly as a function of time, indicating decomposition of the precursor **6** in the reaction mixture, under the alkaline conditions (pH 8.5–9). In the two-step method, the radiolabeling of the SiFA-moiety **5** resulted in incorporation yields ranging from 89 to 99.6 ± 0.5% (*n* = 4), at the optimal time point (2 min) analyzed by radio-TLC. The radiochemical impurities (maximum 11% of total radioactivity) formed in the first step were analyzed by radio-HPLC and are shown in the Supplementary Data (Figure S6). The SiFA radiolabeling was followed by an oxime bond formation between the [^18^F]SiFA ([^18^F]**5**) and tetrazine oxyamine **4** at 42.7 ± 14.2% (*n* = 8) yield in the reaction mixture (Figure S7). Radio-TLC analysis of product [^18^F]**6** revealed the formation of two isomers, with the (*E*)-isomer of [^18^F]**6** being predominant with the amount of the radiolabeled **6** (*Z*)-isomer only 1.45 ± 0.35% (*n* = 16). The final product [^18^F]**6** was isolated at >98% radiochemical purity (Figure S9, radio-TLC) and was subsequently used as a prosthetic group to chemoselectively radiolabel the TCO-functionalized albumin in vitro. The [^18^F]fluorinated albumin [^18^F]**10** was radiolabeled at 99.1 ± 0.2% radiochemical yield (RCY) (*n* = 3) (Figure S12, radio-TLC) and good molar activity (1.1 ± 0.2 GBq/µmol, *n* = 2). To achieve 99% RCY and the total consumption of added [^18^F]**6** (0.26 nmol), minimum of 0.27 nmol of albumin-TCO was to be used. This resulted in 2.5% of the TCOs in the albumin-TCO labeled with [^18^F]**6**. A radiochemical yield of >99% of [^18^F]**10** was achieved by incubating increasing amounts of albumin–TCO with [^18^F]**6** (0.27 nmol) (Figure 1). The radio-HPLC chromatograms of the purified tracers [^18^F]**6** and [^18^F]**10** are presented in the Supplementary Data (Figures S8 and S12).

### 2.3. In Vitro Studies of [^18^F]6 and [^18^F]10.

**Figure 2:**
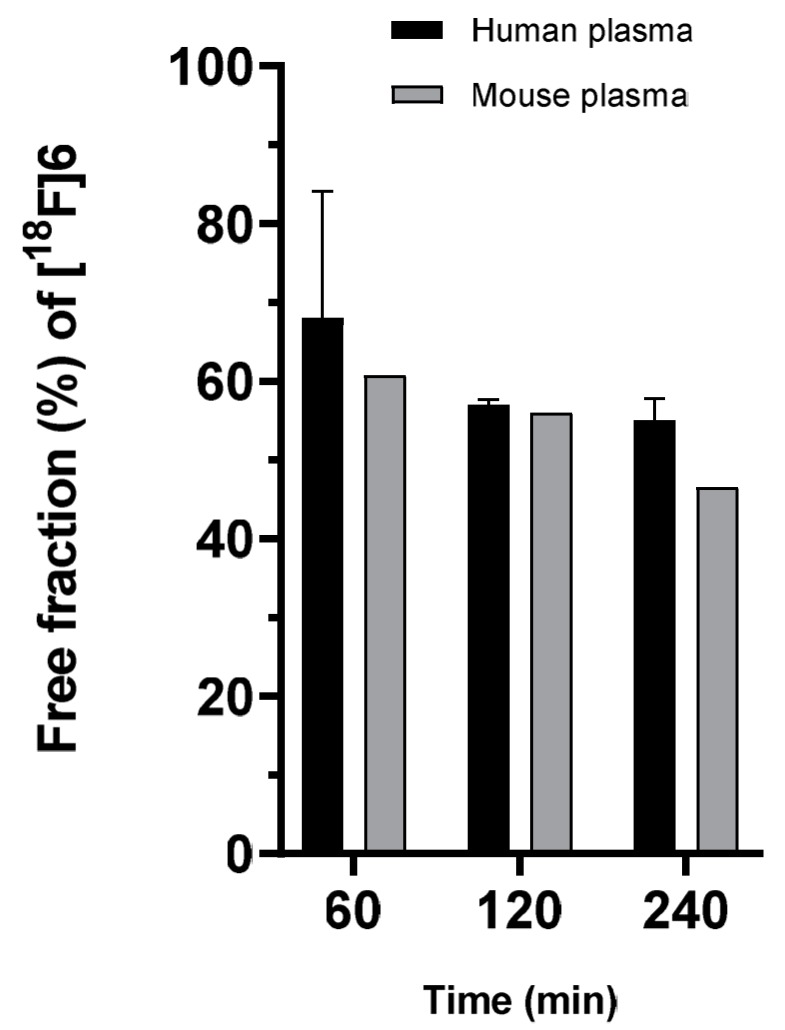
Free fraction of the radiotracer [^18^F]SiFA-Tz ([^18^F]**6**) during 240 min incubation in human and mouse plasma demonstrated comparable profiles with ~50% of the radioactivity in the free fraction at the end of incubation.

The stability of [^18^F]**6** in 0.01M PBS pH 7.4 was shown to be excellent at 90 min, with minimal detachment of fluorine-18 (<1%) during the incubation (Figure S10). Plasma protein binding and metabolic stability of [^18^F]**6** were also evaluated by incubating the radiotracer in plasma and analyzing the deproteinized samples through radio-TLC and radio-HPLC methods, at selected time-points after incubation (Figure 2).

**Figure 3:**
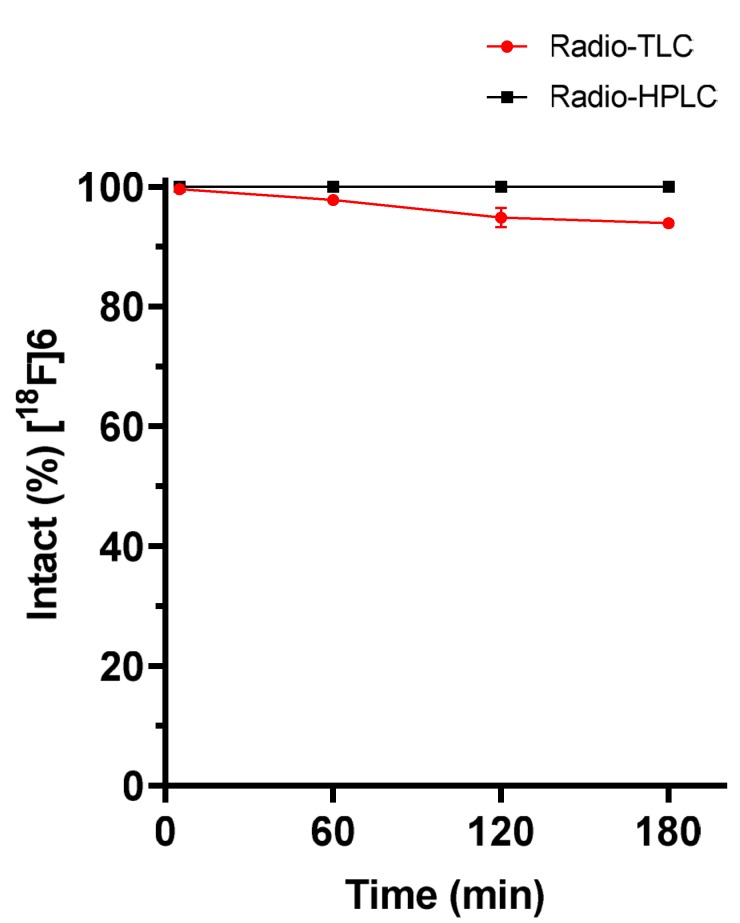
In vitro stability of [^18^F]SiFA-Tz ([^18^F]**6**) after incubation in human plasma at 37 °C, demonstrated excellent stability with ~95% intact tracer at 180 min after start of incubation. The amount of parent [^18^F]**6** was measured through radio-TLC and radio-HPLC analysis.

Through radio-TLC analysis, [^18^F]**6** demonstrated good stability in plasma with up to 6% detachment of the radiolabel over 180 min (94.9 ± 1.6 % intact tracer, *n* = 2). The slight difference in the stability profiles between radio-TLC and radio-HPLC analysis can be explained due to their different ability to quantify free fluoride from the samples. The retention of free fluoride on the silica-based C18 HPLC-column material makes the quantification of free fluoride with HPLC less accurate. Representative radio-TLC and radio-HPLC chromatograms from the experiment are shown in the Supplementary Data (Figures S15 and S16). A radio-HPLC analysis revealed no other radiometabolites of [^18^F]**6** during the 180 min incubation (Figure 3), in addition to a highly polar component, most likely the free fluoride. Despite the observed minor defluorination, the enzymatic stability was found to be sufficient for proceeding into in vivo evaluation.

The Log*D*_pH7.4_ of [^18^F]**6** (1.6 ± 0.2, *n* = 9) was determined by the shake-flask method, as previously reported [16]. It has been shown that lipophilic compounds tend to exhibit a higher plasma-protein binding than the more hydrophilic compounds [29], which support the findings of the measured Log*D*-value and the value obtained for the plasma protein-bound [^18^F]**6**, in this study.

### 2.4. Biodistribution of [^18^F]6 and [^18^F]10

**Figure 4:**
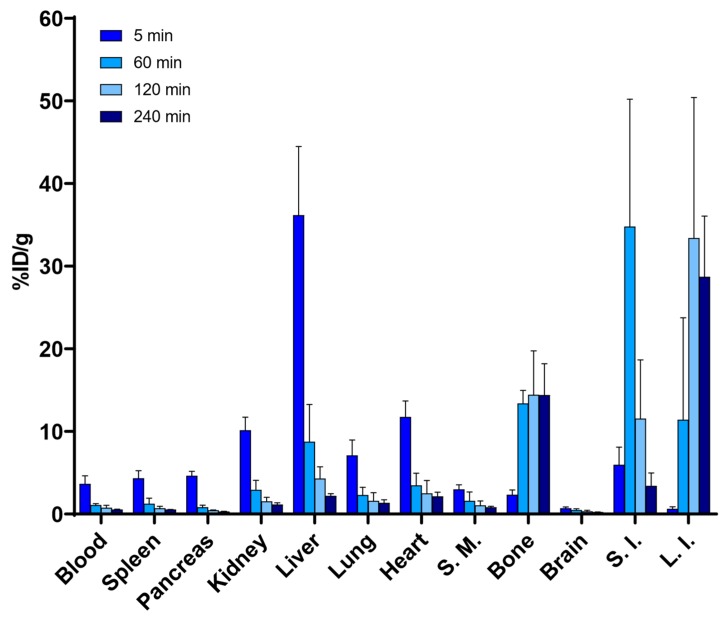
Biodistribution of radioactivity after the intravenous injection of [^18^F]SiFA-Tz ([^18^F]**6**, *n* = 3), demonstrating a high bone uptake of radioactivity, 60 min post-injection. (S.M.—skeletal muscle; bone—tibia, S.I.—small intestine; L.I.—large intestine).

The biodistribution of [^18^F]**6** was investigated in healthy 11 to 12 week-old female CD-1 mice. After intravenous administration into the lateral tail vein, [^18^F]**6** (14.4 ± 0.5 MBq/animal in ~200 µL of 10% EtOH and 0.5% Solutol HS 15 in 0.01 M PBS pH 7.4) exhibited hepatobiliary excretion and a fast clearance from the blood (Figure 4, Figure S13). The highest percentage of the injected dose per gram of tissue (%ID/g) for urine (229.5 ± 204.5) and gallbladder (143.9 ± 103.1) was found to be at 60 min post-injection. In addition to the high radioactivity detected in the urine, gallbladder, liver, and the feces, a high bone uptake of ^18^F^−^ was observed 60 min post-injection (13.4 ± 1.6% ID/g). No major passage of [^18^F]**6** through the blood–brain barrier was observed (0.7 ± 0.2% ID/g) at 5 min post-injection. In order to study the metabolism of [^18^F]**6**, the blood samples were collected at (*t* = 5, 30, and 60 min) post-injection. The deproteinized plasma samples were analyzed through radio-HPLC and radio-TLC methods, which revealed the formation of a highly polar metabolite (R_f_ 0.00 on TLC) that was retained at the origin. On HPLC, a metabolite ([^18^F]**M1**) eluting at 7 min was also detected. Furthermore, there was no indication of more lipophilic metabolites (Figure S17).

**Figure 5:**
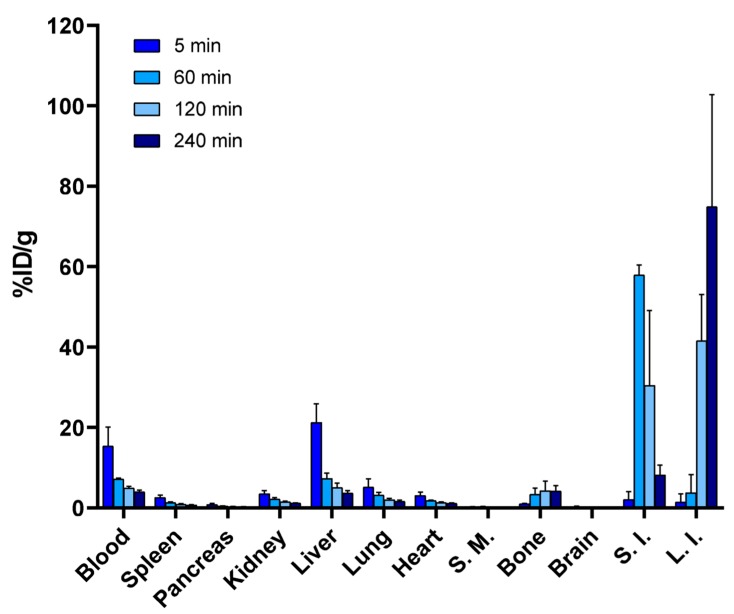
Biodistribution of radioactivity after the intravenous administration of [^18^F]fluoroalbumin. ([^18^F]**10**, *n* = 5) demonstrating a prolonged residence time of radioactivity in whole blood and reduced bone accumulation, indicating resistance to defluorination in vivo. (S.M.—skeletal muscle; bone—tibia; S.I.—small intestine; L.I.—large intestine).

In order to investigate how the conjugation of [^18^F]**6** onto a macromolecule influences its in vivo defluorination rate, we used [^18^F]**6** to synthesize [^18^F]fluoroalbumin ([^18^F]**10**) and evaluated its biodistribution in CD-1 mice. Intravenously injected [^18^F]**10** (0.6 ± 0.1 MBq/animal in ~100 µL of 0.01 M PBS, pH 7.4) had a prolonged residence time in blood with 7.1 ± 0.3% ID/g, at 60-min post-injection and a plasma half-life of 49 min (Figure 5). The highest % ID/g for urine (54.7 ± 25.8) and gallbladder (275.3 ± 185.6), after intravenous injection of [^18^F]**10** was shown to be at 60 min post-injection, for both tissues.

**Figure 6:**
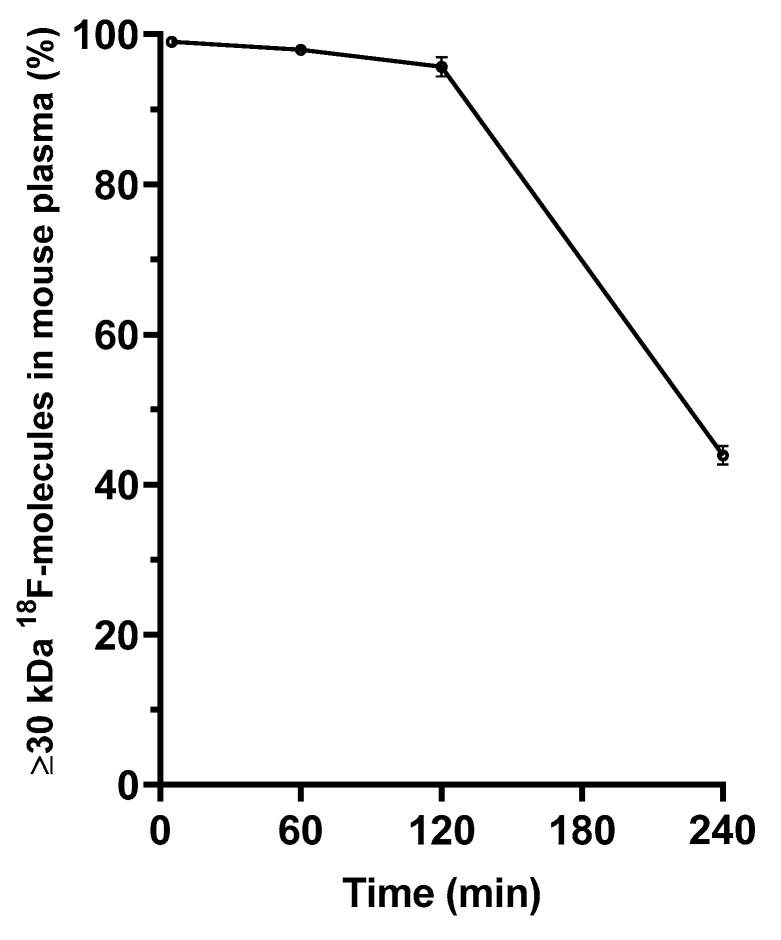
The ex vivo distribution (%) of ≥30 kDa ^18^F-radiolabeled molecules in mouse plasma after intravenous injection of [^18^F]fluoroalbumin ([^18^F]**10**), separated with a molecular weight cut-off filter revealed a highly stable albumin tracer until 2 h post-injection. At 240 min post-injection around 40% of radioactivity was of ≥30 kDa size, with almost 60% of the radioactivity being comprised of species of lower molecular weight.

Stability of [^18^F]**10** was further investigated by separating molecules with molecular weight of ≥30 kDa from plasma samples collected after intravenous administration of [^18^F]**10** through ultrafiltration. The proportion of radiolabeled molecules with a MW of ≥30 kDa (presenting intact [^18^F]**10**) was over 90% until 180 min post-injection, after which it decreased to 40% over the next 60 min (240 min incubation in total), as shown in Figure 6.

**Figure 7:**
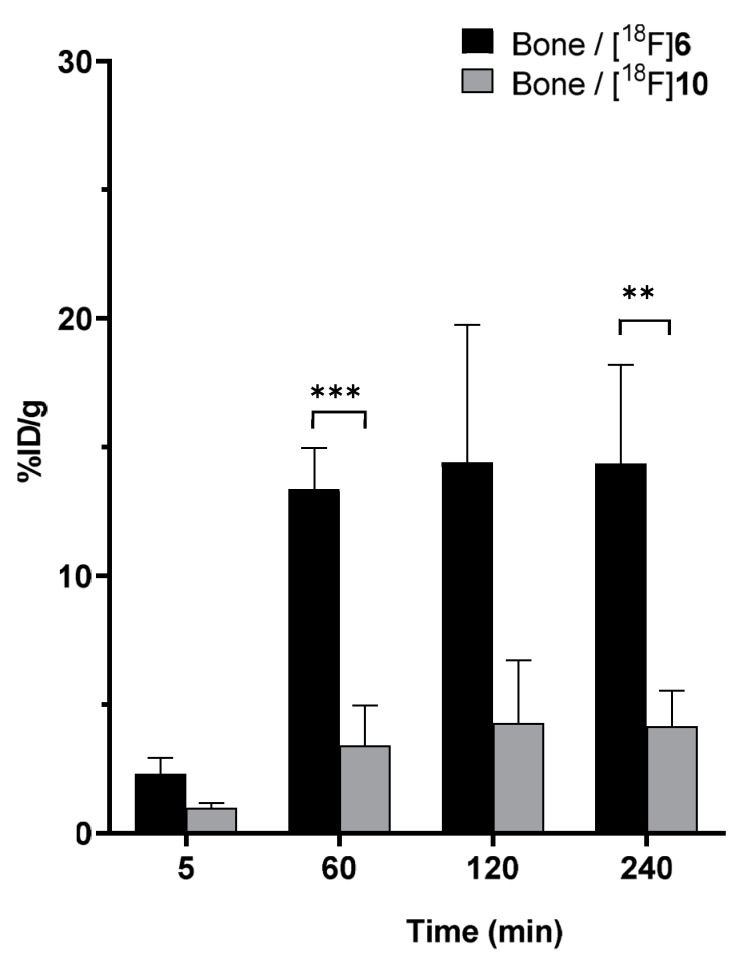
Comparison of the radioactivity accumulated in bone (tibia) in the biodistribution studies of [^18^F]SiFA-Tz ([^18^F]**6**) and [^18^F]fluoroalbumin ([^18^F]**10**). A significant enhancement in stability was seen for the albumin-bound [^18^F]SiFA group in [^18^F]10 (** *p* ≤ 0.01, *** *p* ≤ 0.001), compared to [^18^F]**6**.

By comparing the bone uptake of ^18^F in these two biodistribution studies, it was seen that the ^18^F-Si bond in [^18^F]**10** was noticeably more stable than in the [^18^F]SiFA-Tz ([^18^F]**6**) alone (Figure 7). The bone uptake for both tracers peaked around 120 min after administration and the radioactivity persisted in the bone until the last time point of the biodistribution study, 240 min post-injection. This was an indication of the uptake of free [^18^F]fluoride, which was a result of defluorination of the SiFA moiety, in vivo [30].

**Figure 8:**
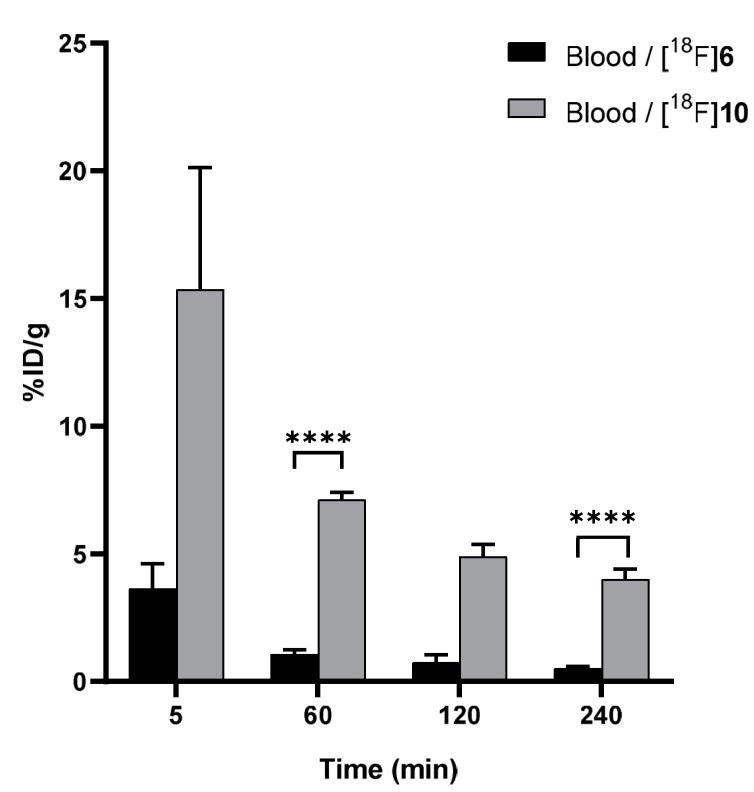
Radioactivity (%ID/g) in ex vivo blood samples at selected time–points, post-injection for [^18^F]SiFA-Tz ([^18^F]**6**) and [^18^F]fluoroalbumin ([^18^F]**10**), demonstrated a significantly prolonged circulation time for the plasma protein tracer ([^18^F]**10**) (**** *p* ≤ 0.0001).

Furthermore, the rate of defluorination was substantially diminished when [^18^F]**6** was used to chemoselectively radiolabel albumin in vitro through the IEDDA ligation. The plasma protein radiotracer [^18^F]**10**, which acted as a model protein in this study, demonstrated a significantly (*p* ≤ 0.0001) longer blood circulation time in healthy CD-1 mice than the ^18^F-labeled tetrazine [^18^F]**6** alone (Figure 8), with a biological half-life similar to what has been reported for other [^18^F]SiFA-radiolabeled serum albumins.

## 3. Discussion

Two PET-tracer candidates, [^18^F]SiFA-Tz ([^18^F]**6**) and [^18^F]fluoroalbumin ([^18^F]**10**) were synthesized and evaluated in vivo. Compound [^18^F]**6** was synthesized with two different synthesis methods, from which the two-step approach was selected, due its higher RCY and good reproducibility. In the one-step radiolabeling approach, the RCY of the product was observed to decrease rapidly as a function of time, indicating decomposition of the precursor in the alkaline reaction mixture. In the two-step method, aminooxy tetrazine **4** was introduced into the reaction mixture at pH 4.6, which was found to be an advantage to avoid any unnecessary decomposition of the tetrazine group. In addition to the two-step method presented in this study, alterative elution protocols with milder reagents, such as copper salts or weak base solutions, as described by Scott et al., should be investigated for the radiolabeling of base sensitive precursors [31]. The in vivo metabolic profile of [^18^F]**6** displayed hepatobiliary elimination, which is characteristic for compounds with low hydrophilicity. Nevertheless, no observable passage of [^18^F]**6** through the blood–brain barrier was detected (0.7 ± 0.2% ID/g) at 5 min post-injection, despite the favorable lipophilicity of the tracer (Log*D* = 1.56 ± 0.20). Based on the radio-HPLC metabolite analysis (Supplement. Figure S17) from ex vivo blood samples and the detection of radioactivity in the bone, we concluded that [**^18^**F]**6** underwent rapid biotransformation, generating highly polar metabolites, one of which was most likely free [**^18^**F]fluoride detached from the radiotracer. Furthermore, the accumulation of radioactivity in bone is a characteristic indication of fast defluorination in vivo. After defluorination, the free fluoride is sequestered rapidly from circulation and either binds into the surface of the bone or accumulates irreversibly into the hydroxyapatite Ca_10_(PO_4_)_6_(OH)_2_, forming fluorapatite (Ca_10_(PO_4_)_6_F_2_) [30]. Thus, it was evident that unexpected and relatively fast defluorination was observed in vivo. Free fluoride was also excreted into the urine, in vivo. Defluorination of ^18^F-radiolabeled tracer [^18^F]**6** could be detected in the bone as early as 10–20 min after injection [30]. The observed rapid defluorination in vivo limited the utility of [^18^F]**6** for pretargeted PET imaging, and further structural optimization was warranted to stabilize the structure towards the defluorination. However, since the stability of biomacromolecular SiFA conjugates has been reported to be good, the possibility of using [^18^F]**6** as a prosthetic group for the in vitro bioorthogonal radiolabeling of proteins was investigated through administration of [^18^F]**10**, to healthy mice. Stability of the [^18^F]SiFA-Tz group against in vivo defluorination was dramatically improved when the group was bound to albumin (13.4 ± 1.6% ID/g in bone for [^18^F]**6** vs. 3.4 ± 1.5% ID/g in bone for [^18^F]**10**, at 60 min post-injection). Furthermore, the blood circulation half-life was 48 min, which is in the order of the reported plasma half-life of 60 min, for the bovine serum albumin in mice [32,33].

There are some examples of small molecular SiFA derivatives and a SiFA-conjugated peptide that have exhibited detectable in vivo defluorination, but not at the level observed in our study [34,35]. Rat serum albumin (RSA) radiolabeled with [^18^F]SiFA, through isothiocyanate modification of lysine residues has been shown to be relatively stable with only a low rate of defluorination, until 90 min after administration [36]. It has also been shown that the conjugation position of the [^18^F]SiFA-moiety on the albumin could have an influence on the rate of defluorination in vivo. A more stable maleimido-[^18^F]SiFA conjugated to RSA via thiol groups is an example of the enhanced stability of the radiolabel in a [^18^F]SiFA-radiolabeled serum albumin [37]. Thus, this radiolabeling system could be further improved by using a more selective conjugation chemistry (maleimide over N-hydroxysuccinimide) for the addition of the TCO to albumin, while simultaneously optimizing the TCO:albumin ratio and availability of the TCO moiety to the IEDDA reaction, with [^18^F]**6**. Nevertheless, our results demonstrated the feasibility of using the highly selective and rapid bioorthogonal reaction strategy for the radiolabeling of biomacromolecules with fluorine-18, under mild reaction conditions.

## 4. Materials and Methods

All reagents and solvents were purchased from commercial providers and used as received without further purification. Hyox-18 ^18^O–enriched water (98%) was purchased from Rotem Industries Limited (Arava, Israel). Ultrapure water (18.0 MΩ) was produced with a Milli-Q Integral Water Purification System (Merck Millipore, Burlington, MA, USA). HATU, DMF, DIPEA, DMSO, LiCl, methanol, aniline, Kryptofix 2.2.2, 1 M HCl in diethylether, formic acid, ethylacetate and boc-aminooxy acetic acid were purchased from Sigma-Aldrich (St. Louis, MO, USA). MgSO4 was purchased from Merck Millipore (Darmstadt, Germany). TCO-PEG4-NHS ester was purchased from either Jena Biosciences (Jena, Germany) or Conju-Probe (San Diego, CA, USA). Tetrazine amine was purchased from either BroadPharm (San Diego, CA, USA) or Conju-Probe (San Diego, CA, USA). DNA synthesis quality anhydrous acetonitrile (max. 10 ppm water) was purchased from Merck (Kenilworth, NJ, USA). SiFA-aldehyde was purchased from Enamine (Monmouth, NJ, USA). Bovine serum albumin was purchased from Merck (Kenilworth, NJ, USA). Moisture or air sensitive reactions were carried out under an argon atmosphere in oven-dried glassware. Reactions were monitored by TLC Silica gel 60 F254 Merck Millipore (Darmstadt, Germany). Silica gel TLC-plates were run in EtOAc:heptane (7:3) as eluent. [^18^F]SiFA-Tz ([^18^F]**6**) R*_f_* = 0.59, [^18^F]fluoroalbumin ([^18^F]**10**) R*_f_* = 0.00.

^1^H-, ^13^C-, and ^19^F-NMR spectra were acquired with a Varian Mercury spectrometer (300 MHz, 500 MHz, 600 MHz) (Palo Alto, CA, USA). Chemical shifts (δ) are reported in ppm units, using the solvent residual signal as a reference. Coupling constants (*J*) are expressed in hertz (Hz). The purities of radiolabeled compounds were determined through RP-HPLC with photodiode array (PDA)-, and radiodetector and through silica TLCs analyzed with a Fujifilm FLA 5100 scanner (Fujifilm Life sciences, Cambridge, MA, USA). The excised tissue samples were measured with 1480 Wallac Wizard^®^ 3” (PerkinElmer^TM^ Life Sciences, Waltham, MA, USA) gamma counter for 60 s per sample.

High performance liquid chromatography was carried out with a Shimadzu HPLC system consisting of a DGU-20A degasser, an LC-20AD UPLC LC unit, a SIL-20A HT autosampler, a CTO20 AC column oven, a CBM-20A communications bus module, a Scionix Holland scintillation detector with a 51 BP 51/2 NaI(Tl) crystal and an SPD-M20A diode array detector. For the [^18^F]SiFATz, a Waters Symmetry semi-preparative C18 column (300 × 7.8 mm, 7 µm) was used, with 0.01 M H_3_PO_4_:ACN (20:80, 3 mL/min) as the eluent. Phenomenex BioSep SEC s3000 size exclusion column was used, with 0.1 M phosphate buffer pH 7 (0.8 mL/min) as the eluent, to analyze the conjugated albumin-TCO and radiolabeled protein tracer [^18^F]fluoroalbumin ([^18^F]10).

Preparative high performance liquid chromatography was carried out using a system consisting of a Phenomenex Degassex^TM^ DG-4400 degasser, Merck LaChrom L-7100 pump, in-house prepared remote-controlled injection system, Amersham pharmacia biotech REC 112 dual channel chart recorder, Carroll & Ramsey Associates 101-H-DC3 multi-channel radiation detector, and a Knauer Azura UVD 2.1S detector. A waters Symmetry semi-preparative C18 column (300 × 7.8 mm, 7 µm), with 0.01 M H3PO4:ACN (20:80, 3 mL/min flowrate) as the eluent was used for the preparative purification of the [^18^F]SiFA-Tz radiotracer ([^18^F]6).

### 4.1. Synthesis

*Tert-butyl(2-{[4-(1,2,4,5-tetrazin-3-yl)ben-zyl]amino}-2-oxoethoxy)carbamate* (**3**). HATU (77 mg, 201 µmol) in DMF (0.5 mL) was added to a solution of **1** (32 mg, 168 µmol) in dry DMF (0.7 mL), under argon. The mixture was stirred at room temperature for 10 min, then **2** (25 mg, 112 µmol) in DMF (2.5 mL) was added, followed by DIPEA (49 µL, 281 µmol). After 20 h mixing at room temperature, ethyl acetate (15 mL) was added and the organic phase was extracted with ultrapure water (10 mL). The organic phase was extracted with 5% LiCl solution (3 × 10 mL, 1 × 20 mL), dried over MgSO4, and concentrated in vacuo. The crude product was purified through silica gel column chromatography (EtOAc:heptane) to afford **3** as a pink solid (26 mg, 72 µmol, 65% yield).

^1^H-NMR (300 MHz, CDCl_3_, Supplement Figure S1) δ 10.20 (d, *J* = 0.6 Hz, 1H), 8.79 (s, 1H), 8.61–8.49 (m, 2H), 7.72 (s, 1H), 7.60–7.51 (m, 2H), 4.63 (d, *J* = 6.1 Hz, 2H), 4.40 (s, 2H), 1.42 (d, *J* = 0.6 Hz, 9H). ^13^C-NMR (75 MHz, CDCl3, Supplement Figure S2) 169.29, 166.53, 158.19, 157.98, 144.09, 130.73, 128.00, 83.63, 68.00, 42.83, 28.27 ppm.

*N-[4-(1,2,4,5-tetrazin-3-yl)benzyl]-2-(aminooxy)acetamide hydrochloride* (**4**). 1 M HCl in diethyl ether (50 mL) was added to a solution of **3** (22 mg, 61 µmol) in MeOH (4 mL). After 24 h, the solution was concentrated in vacuo, to afford **4** as a pink solid. The solid was dissolved in 2 mL of methanol and 20 mL of cold diethyl ether was added to the solution. The closed flask was kept in +4 °C overnight, to facilitate the crystallization of the purified product. The recrystallized solid (10 mg, 39 µmol, 65% yield) was suction filtered and used as such in the next reaction step.

*(E,Z)-N-[4-(1,2,4,5-tetrazin-3-yl)benzyl]-2-({[4-(di-tert-butylfluorosilyl)benzylidene]-amino}oxy)acetamide* (**6**). Compound **5** (8.5 mg, 32 µmol) in ACN (0.5 mL) was added into a solution of **4** (4 mg, 15 µmol) in 0.3 M anilinium acetate buffer pH 4.6 (2 mL). After 15 min, ultrapure water (10 mL) was added to the reaction mixture, concentrated with two Sep-pak C18 Light cartridges (Milford, MA, USA) and eluted with ACN (5 mL). The crude product was purified through RP-HPLC (0.01 M H3PO4:CAN, 20:80, 3 mL/min) to afford **6** as a pink solid (7 mg, 14 µmol, 90% yield). 

^1^H-NMR (300 MHz, CDCl3, Supplement Figure S3) δ 10.21 (s, 1H), 8.57 (d, *J* = 8.5 Hz, 2H), 8.21 (s, 1H), 7.69–7.49 (m, 6H), 4.76 (s, 2H), 4.68 (d, *J* = 6.1 Hz, 2H), 1.05 (d, *J* = 1.1 Hz, 18H). ^19^F-NMR (282 MHz, CDCl3 Supplement Figure S4) δ-189.13. ^13^C-NMR (126 MHz, CDCl_3_, Supplement Figure S5) δ 170.10, 166.58, 158.15, 151.49, 143.92, 134.80, 134.77, 132.32, 131.18, 129.02, 128.65, 126.67, 77.61, 77.56, 77.36, 77.11, 73.68, 42.93, 27.61, 20.64, 20.54. ESI-TOF MS: Calculated for C_26_H_34_FN_6_O_2_Si [M + H]^+^ 509.24911 *m*/*z*, found 509.2147 *m*/*z*. Calculated [M + Na]^+^ 531.22378 *m*/*z*, found 531.1967 *m*/*z*. Calculated [M + K]^+^ 547.19771 *m*/*z*, found 547.1706 *m*/*z*.

Albumin trans-cyclooctene (albumin-TCO (**9**)). TCO-PEG4-NHS ester (5 mg, 9.7 µmol) in DMSO : 0.5 M borate buffer (1:1, 1 mL) pH 9 was added to a solution of bovine serum albumin **7** (3.5 mg, 53 nmol) in borate buffer pH 9.0 (1 mL). After 1 h, the reaction mixture was purified with a PD-10 size-exclusion column (GE Healthcare, Chicago, IL, USA), using ultrapure water as the eluent. The collected fractions were analyzed by a SEC-column HPLC, using 0.1 M PBS as an eluent, with a flow rate of 0.8 mL/min, for identifying the fractions containing albumin-TCO. The fractions containing albumin-TCO were lyophilized to afford **9** as white solid (*n* = 3). Albumin-TCO (1 mg) was dissolved in 1 mL of ultrapure water and analyzed with MALDI-TOF-MS (calculated for bovine serum albumin 66,338 Da, measured for albumin-TCO 82039-82265 Da).

### 4.2. Radiochemistry

No-carrier-added ^18^F-Fluoride was produced in-house with Cyclone 10/5 cyclotron (IBA, Louvain-la-Neuve, Belgium) through a ^18^O(*p,n*)^18^F nuclear reaction, by bombarding H_2_^18^O with 10 MeV protons. The radiosynthesis was carried out in a semiautomatic synthesis unit (DM Automation), with an integrated preparative HPLC system for the purification of the radiotracer. The nucleophilic ^18^F^–^ was trapped on a Waters QMA Light ion-exchange cartridge, followed by elution with a basic K[^18^F]FK2.2.2-complex solution. Water residue was evaporated azeotropically by adding anhydrous ACN, followed by heating, under a 40-mL/min argon flow.

#### 4.2.1. Radiosynthesis of [^18^F]6

In the one-step method, precursor 6, dissolved in 500 µL of anhydrous acetonitrile, was added into the dried K[^18^F]F/K2.2.2 and incubated for 2 min (25 °C). The reaction mixture was diluted with an additional 500 µL of anhydrous acetonitrile for the radio-TLC and radio-HPLC analysis.

In the two-step method, after evaporation of the solvent, SiFA (**5**) in anhydrous ACN (0.5 mL) was added into the reaction vial containing K[^18^F]F/K2.2.2 and incubated at room temperature for 2 min. Tetrazine oxyamine (**3**) in 0.3 M anilinium acetate buffer pH 4.6 (200 µL) and ACN (50 µL) was added into the reaction mixture, and the reaction was further incubated at room temperature, for 15 min (RCY 74%, radio-TLC), and purified by preparative HPLC, providing 385 MBq of [^18^F]**6** at 115 min from end-of-bombardment (EOB, 14.8 ± 1.6% DCY) and 5 GBq/µmol at end-of-bombardment (EOS). The purified (RCP 98.8 ± 1.3%, *n* = 16) product [^18^F]**6** was formulated (10% ethanol, 0.5% Solutol HS 15 in 0.01 M PBS), sterile filtered (0.22 µm), and used as such for injections.

#### 4.2.2. Radiosynthesis of [^18^F]10

The formulated [^18^F]**6** was added onto the lyophilized albumin-TCO (350 µg) and incubated at room temperature for 15 min. The produced [^18^F]fluoroalbumin ([^18^F]10) was purified by centrifugation (10,000 *g*, Eppendorf Centrifuge 5430, Ag, Hamburg, Germany) with molecular weight cut-off (MWCO) filters (30K, VWR^®^, Radnor, PA, USA), with 0.01 M PBS as the eluent, sterile-filtered and used as such for injections. The apparent molar activity was 1.1 GBq/µmol of protein at EOS. The radio-HPLC chromatogram of the purified [^18^F]**10** is presented in the Supplementary Data (Figure S11).

### 4.3. In Vitro Experiments

#### 4.3.1. Log*D*_pH7.4_ Determination for [^18^F]6

A total of 25 µL of [^18^F]**6** was added to a mixture of 1-octanol and 0.02 M PBS (pH 7.4) in a 1.5 mL microtube. The mixture was shaken mechanically (500 rpm) for 10 min and centrifuged (1000 g, 5 min), and the layers were separated. A sample of 500 µL of each layer were pipetted into pre-weighed polypropylene tubes and the activity in the samples was measured with a Wizard gamma counter. The distribution of [^18^F]**6** between octanol and PBS was calculated, according to

$$\mathrm{Log}D_{7.4}=Log\;\frac{Ac_{OCT}}{Ac_{PBS}}$$

where Ac*oct* = activity concentration of octanol and Ac*_PBS_* = activity concentration of PBS. Log*D*_pH7.4_ = 1.56 ± 0.20 (*n* = 5).

#### 4.3.2. In Vitro Stability and Plasma Protein Binding for [^18^F]6

A total of 40 µL of [^18^F]**6** was incubated in 0.01 M PBS at room temperature, in a microtube for 90 min, with mixing (400 rpm). At selected time-points (*t* = 5, 30, 60 and 90 min, *n* = 1), the samples were injected into an HPLC with PDA- and radiodetector, for analysis. Radiotracer [^18^F]**6** (40 µL) was incubated in 50% human plasma (anonymous donor FFP-24 plasma provided by the Finnish Red Cross Blood Service, Helsinki, Finland) in PBS at 37 °C, and in mouse plasma (separated from CD-1 mouse blood). At selected time-points (5, 60, 120, and 240 min, *n* = 2 for each), 100 µL of the samples were taken, diluted with 200 µL of cold acetonitrile, and centrifuged at 10,000 g for 5 min. The radioactivity in the precipitated pellet (protein-bound fraction) and supernatant (free fraction) were measured with a gamma counter and 100 µL of the samples were injected into HPLC, for radio-HPLC analysis.

### 4.4. Biological Studies

All animal experiments were conducted under a project license approved by the National Board of Animal Experimentation in Finland (license number ESAVI/12132/04.10.07/2017). The animals were group-housed in standard polycarbonate cages, on aspen bedding, in a HEPA-filtered housing unit (UniProtect, Ehret, Emmendingen, Germany), with food (Envigo Teklad Global Diet 2016) and tap water, available ad libitum. Conditions were maintained at 21 ± 1 °C and 55 ± 15% relative humidity, with a 12:12 lighting cycle. The biodistribution studies were conducted in healthy, female CD-1 mice (weight 25–33 g, 11 to 12 weeks, Charles River). The radiotracers [^18^F]**6** and [^18^F]**10** were injected via the tail vein to CD-1 mice, in the following formulations—10% EtOH and 0.5% Solutol HS 15 in 0.01 M PBS pH 7.4 for [^18^F]6 and 0.01 M PBS pH 7.4 for [^18^F]10. At selected time-points (5, 60, 120, and 240 min), the mice were euthanized with CO2 asphyxiation, followed by cervical dislocation, and selected tissues were collected, weighed, and the radioactivity was measured on an automated gamma counter.

#### 4.4.1. Biodistribution of [^18^F]6

14.3 ± 0.5 MBq (*n* = 12) (25.4 ± 1.4 µg, 44.2 ± 2.4 nmol) of 96.2% pure [^18^F]6 was injected into the tail vein of healthy female CD-1 mice (*n* = 3 per time-point), to evaluate the biodistribution and stability of the tracer in vivo. The mice were euthanized at selected time-points (*t* = 5, 60, 120, and 240 min) and the tissues were collected and measured with a gamma counter, as described above. For metabolite studies, blood from a cardiac puncture was collected into an Eppendorf tube containing 2 µL of 1% heparin solution in 0.9% NaCl (aq.) and centrifuged at 1000 *g*, for 10 min, to separate the plasma from the blood cells. Cold acetonitrile (twice the volume of separated plasma) was added into the plasma and centrifuged (at 10 000 g for 5 min) to precipitate the proteins. A small sample (4 µL) was applied onto a silica TLC and analyzed with digital autoradiography.

#### 4.4.2. Biodistribution of [^18^F]10

A total of 0.6 ± 0.1 MBq (*n* = 15) (43.2 ± 1.4 µg of protein) of [^18^F]fluoroalbumin was injected into the tail vein of female CD-1 mice (*n* = 3–4 per time point). The mice were euthanized at selected time-points and the tissues were collected and measured with a gamma counter, as described above. Blood from a cardiac puncture was collected into an Eppendorf tube containing 2 µL of 1% heparin solution in 0.9% NaCl (aq) and was pretreated before analysis, as described above. After centrifugation, a small sample (4 µL) was applied onto a silica TLC plate and 100 µL of the supernatant was injected into HPLC for radiometabolite analysis. For the radiometabolite studies of [^18^F]**10**, the blood was collected into an 1.5-mL microtube containing 1% heparin solution in 0.9% NaCl (aq), and was centrifuged (at 1000 *g* for 10 min). Plasma was separated from the cell pellet and added onto a 30-kDa molecular weight cut-off (MWCO) centrifugal filter (VWR^®^ Radnor, PA, USA) and centrifuged (at 6500 rpm for 10 min). The filter with over 30 kDa ^18^F-labeled molecules and the microtube containing the below-30-kDa ^18^F-labeled molecules in the eluate, were measured with a gamma counter, to determine the percentage of small molecular weight metabolites from over 30 kDa molecules representing the intact BSA in the plasma.

#### 4.4.3. Statistical Analysis

Statistical significance of ^18^F bone accumulation after intravenous administration and the tracer blood circulation time were analyzed using an unpaired *t*-test (GraphPad Prism 8.0.1). The values presented in the synthesis, biodistribution studies, and Log*D-*measurements are mean ± standard deviation.

## 5. Conclusions

Despite of promising hydrolytic stability in vitro, [^18^F]SiFA-Tz ([^18^F]**6**) demonstrated fast defluorination in vivo, after intravenous administration in CD-1 mice limiting its utility as a standalone radiotracer, for pretargeted PET imaging. However, the fluorine-18 label in the biomacromolecular radiotracer [^18^F]fluoroalbumin ([^18^F]**10**), which was radiolabeled as a proof-of-concept model compound with [^18^F]**6**, was found to be metabolically more stable, suggesting the utility of [^18^F]SiFA-Tz as a prosthetic group for in vitro radiolabeling of biomolecules of higher molecular weight. Based on these findings, the structure of [^18^F]SiFA-Tz warrants further optimization, before it can be considered for use as a radiolabeling tool for low molecular weight biomolecules or as a tracer for pretargeted PET imaging.

## Supplementary Materials

The following are available online, NMR spectra, HPLC chromatograms, autoradiography profiles, and biodistributions of synthesized and radiolabeled compounds. Supplementary data are provided as a separate .pdf document.

## References

[1] Jacobson O., Kiesewetter D.O., Chen X. (2015). Fluorine-18 radiochemistry, labeling strategies and synthetic routes. Bioconjug. Chem..

[2] Deng X., Rong J., Wang L., Vasdev N., Zhang L., Josephson L., Liang S.H. (2019). Chemistry for Positron Emission Tomography: Recent Advances in ^11^C-, ^18^F-, ^13^N-, and ^15^O-Labeling Reactions. Angew. Chem. Int. Ed. Engl..

[3] Preshlock S., Tredwell M., Gouverneur V. (2016). ^18^F-Labeling of Arenes and Heteroarenes for Applications in Positron Emission Tomography. Chem. Rev..

[4] Brooks A.F., Topczewski J.J., Ichiishi N., Sanford M.S., Scott P.J. (2014). Late-stage [^18^F]Fluorination: New Solutions to Old Problems. Chem. Sci..

[5] Oliveira B.L., Guo Z., Bernardes G.J.L. (2017). Inverse electron demand Diels-Alder reactions in chemical biology. Chem. Soc. Rev..

[6] Rostovtsev V.V., Green L.G., Fokin V.V., Sharpless K.B. (2002). A stepwise huisgen cycloaddition process: Copper(I)-catalyzed regioselective “ligation” of azides and terminal alkynes. Angew. Chem. Int. Ed. Engl..

[7] Tornoe C.W., Christensen C., Meldal M. (2002). Peptidotriazoles on solid phase: [1,2,3]-triazoles by regiospecific copper(I)-catalyzed 1,3-dipolar cycloadditions of terminal alkynes to azides. J. Org. Chem..

[8] Agard N.J., Prescher J.A., Bertozzi C.R. (2004). A strain-promoted [3 + 2] azide-alkyne cycloaddition for covalent modification of biomolecules in living systems. J. Am. Chem. Soc..

[9] Carboni R.A., Lindsey R.V. (1959). Reactions of Tetrazines with Unsaturated Compounds - a New Synthesis of Pyridazines. J. Am. Chem. Soc..

[10] Edem P.E., Sinnes J.P., Pektor S., Bausbacher N., Rossin R., Yazdani A., Miederer M., Kjaer A., Valliant J.F., Robillard M.S. (2019). Evaluation of the inverse electron demand Diels-Alder reaction in rats using a scandium-44-labelled tetrazine for pretargeted PET imaging. Ejnmmi. Res..

[11] Houghton J.L., Membreno R., Abdel-Atti D., Cunanan K.M., Carlin S., Scholz W.W., Zanzonico P.B., Lewis J.S., Zeglis B.M. (2017). Establishment of the In Vivo Efficacy of Pretargeted Radioimmunotherapy Utilizing Inverse Electron Demand Diels-Alder Click Chemistry. Mol. Cancer Ther..

[12] Lappchen T., Rossin R., van Mourik T.R., Gruntz G., Hoeben F.J.M., Versteegen R.M., Janssen H.M., Lub J., Robillard M.S. (2017). DOTA-tetrazine probes with modified linkers for tumor pretargeting. Nucl. Med. Biol..

[13] Membreno R., Cook B.E., Fung K., Lewis J.S., Zeglis B.M. (2018). Click-Mediated Pretargeted Radioimmunotherapy of Colorectal Carcinoma. Mol. Pharm..

[14] Meyer J.P., Houghton J.L., Kozlowski P., Abdel-Atti D., Reiner T., Pillarsetty N.V., Scholz W.W., Zeglis B.M., Lewis J.S. (2016). ^18^F-Based Pretargeted PET Imaging Based on Bioorthogonal Diels-Alder Click Chemistry. Bioconjug. Chem..

[15] Li Z., Cai H., Hassink M., Blackman M.L., Brown R.C., Conti P.S., Fox J.M. (2010). Tetrazine-trans-cyclooctene ligation for the rapid construction of ^18^F-labeled probes. Chem. Commun. (Camb).

[16] Fersing C., Bouhlel A., Cantelli C., Garrigue P., Lisowski V., Guillet B. (2019). A Comprehensive Review of Non-Covalent Radiofluorination Approaches Using Aluminum [^18^F]fluoride: Will [^18^F]AlF Replace ^68^Ga for Metal Chelate Labeling?. Molecules.

[17] Keinänen X.G., Li N.K., Chenna D., Lumen J., Ott C.F.M., Molthoff M., Sarparanta K., Helariutta T., Vuorinen A.D., Windhorst A.J. (2016). New Highly Reactive and Low Lipophilicity Fluorine18 Labeled Tetrazine Derivative for Pretargeted PET Imaging. ACS Med. Chem. Lett..

[18] Schirrmacher E., Wangler B., Cypryk M., Bradtmoller G., Schafer M., Eisenhut M., Jurkschat K., Schirrmacher R. (2007). Synthesis of p-(di-tert-butyl[^18^F]fluorosilyl)benzaldehyde ([^18^F]SiFA-A) with high specific activity by isotopic exchange: A convenient labeling synthon for the ^18^F-labeling of N-aminooxy derivatized peptides. Bioconjug. Chem..

[19] Berke S., Kampmann A.L., Wuest M., Bailey J.J., Glowacki B., Wuest F., Jurkschat K., Weberskirch R., Schirrmacher R. (2018). ^18^F-Radiolabeling and In Vivo Analysis of SiFA-Derivatized Polymeric Core-Shell Nanoparticles. Bioconjug. Chem..

[20] Niedermoser S., Chin J., Wangler C., Kostikov A., Bernard-Gauthier V., Vogler N., Soucy J.P., McEwan A.J., Schirrmacher R., Wangler B. (2015). In Vivo Evaluation of ^18^F-SiFAlin-Modified TATE: A Potential Challenge for ^68^Ga-DOTATATE, the Clinical Gold Standard for Somatostatin Receptor Imaging with PET. J. Nucl. Med..

[21] Wangler B., Quandt G., Iovkova L., Schirrmacher E., Wangler C., Boening G., Hacker M., Schmoeckel M., Jurkschat K., Bartenstein P. (2009). Kit-like ^18^F-labeling of proteins: Synthesis of 4-(di-tert-butyl[^18^F]fluorosilyl)benzenethiol (Si[^18^F]FA-SH) labeled rat serum albumin for blood pool imaging with PET. Bioconjug. Chem..

[22] Wangler C., Waser B., Alke A., Iovkova L., Buchholz H.G., Niedermoser S., Jurkschat K., Fottner C., Bartenstein P., Schirrmacher R. (2010). One-step ^18^F-labeling of carbohydrate-conjugated octreotate-derivatives containing a silicon-fluoride-acceptor (SiFA): In vitro and in vivo evaluation as tumor imaging agents for positron emission tomography (PET). Bioconjug. Chem..

[23] Zhu J., Chin J., Wangler C., Wangler B., Lennox R.B., Schirrmacher R. (2014). Rapid ^18^F-labeling and loading of PEGylated gold nanoparticles for in vivo applications. Bioconjug. Chem..

[24] Zhu J., Li S., Wangler C., Wangler B., Lennox R.B., Schirrmacher R. (2015). Synthesis of 3-chloro-6-((4-(di-tertbutyl[^18^F]fluorosilyl)-benzyl)oxy)-1,2,4,5-tetrazine ([^18^F]SiFA-OTz) for rapid tetrazine-based ^18^F-radiolabeling. Chem. Commun..

[25] Keinänen O., Partelova D., Alanen O., Antopolsky M., Sarparanta M., Airaksinen A.J. (2018). Efficient cartridge purification for producing high molar activity [^18^F]fluoro-glycoconjugates via oxime formation. Nucl. Med. Biol..

[26] Keinänen O., Fung K., Pourat J., Jallinoja V., Vivier D., Pillarsetty N.K., Airaksinen A.J., Lewis J.S., Zeglis B.M. (2017). Sarparanta, M. Pretargeting of internalizing trastuzumab and cetuximab with a ^18^F-tetrazine tracer in xenograft models. EJNMMI Res..

[27] Keinänen O., Mäkilä E.M., Lindgren R., Virtanen H., Liljenback H., Oikonen V., Sarparanta M., Molthoff C., Windhorst A.D., Roivainen A. (2017). Pretargeted PET Imaging of trans-Cyclooctene-Modified Porous Silicon Nanoparticles. ACS Omega.

[28] Kolmel D.K., Kool E.T. (2017). Oximes and Hydrazones in Bioconjugation: Mechanism and Catalysis. Chem. Rev..

[29] Fauber B.P., Rene O., Boenig G.d.L., Burton B., Deng Y., Eidenschenk C., Everett C., Gobbi A., Hymowitz S.G. (2014). Reduction in lipophilicity improved the solubility, plasma-protein binding, and permeability of tertiary sulfonamide RORc inverse agonists. Bioorg. Med. Chem. Lett..

[30] Piert M., Zittel T.T., Becker G.A., Jahn M., Stahlschmidt A., Maier G., Machulla H.J., Bares R. (2001). Assessment of porcine bone metabolism by dynamic. J. Nucl. Med..

[31] Mossine A.V., Brooks A.F., Ichiishi N., Makaravage K.J., Sanford M.S., Scott P.J. (2017). Development of Customized [^18^F]Fluoride Elution Techniques for the Enhancement of Copper-Mediated Late-Stage Radiofluorination. Sci. Rep..

[32] Kallinen A.M., Sarparanta M.P., Liu D.F., Mäkilä E.M., Salonen J.J., Hirvonen J.T., Santos H.A., Airaksinen A.J. (2014). In Vivo Evaluation of Porous Silicon and Porous Silicon Solid Lipid Nanocomposites for Passive Targeting and Imaging. Mol. Pharmaceut..

[33] Matsumura Y., Maeda H. (1986). A new concept for macromolecular therapeutics in cancer chemotherapy mechanism of tumoritropic accumulation of proteins and the antitumor agent smancs. Cancer Res..

[34] Dialer L.O., Selivanova S.V., Muller C.J., Muller A., Stellfeld T., Graham K., Dinkelborg L.M., Kramer S.D., Schibli R., Reiher M. (2013). Studies toward the development of new silicon containing building blocks for the direct ^18^F-labeling of peptides. J. Med. Chem..

[35] Lindner S., Michler C., Leidner S., Rensch C., Wangler C., Schirrmacher R., Bartenstein P., Wangler B. (2014). Synthesis and in vitro and in vivo evaluation of SiFA-tagged bombesin and RGD peptides as tumor imaging probes for positron emission tomography. Bioconjug. Chem..

[36] Rosa-Neto P., Wangler B., Iovkova L., Boening G., Reader A., Jurkschat K., Schirrmacher E. (2009). [^18^F]SiFA isothiocyanate: A new highly effective radioactive labeling agent for lysine-containing proteins. Chembiochem.

[37] Iovkova L., Wangler B., Schirrmacher E., Schirrmacher R., Quandt G., Boening G., Schurmann M., Jurkschat K. (2009). para-Functionalized aryl-di-tert-butylfluorosilanes as potential labeling synthons for ^18^F-radiopharmaceuticals. Chemistry.

