# Supplementary material for: Evaluation of Organo [18F]Fluorosilicon Tetrazine as a Prosthetic Group for the Synthesis of PET Radiotracers
Source: Molecules. 2020 Mar 7;25(5):1208. doi: 10.3390/molecules25051208 (PMC7179430; doi:10.3390/molecules25051208)
Supplement: Supplementary file 1 [file molecules-25-01208-s001.pdf]

SUPPLEMENTARY DATA

Evaluation of Organo[^{18}F]fluorosilicon Tetrazine as a Prosthetic Group for Synthesis of PET Radiotracers

Sofia Otaru[†], Surachet Imlimthan[†], Mirkka Sarparanta[†], Kerttuli Helariutta[†], Kristiina Wähälä[†,§], Anu J. Airaksinen[†,¤,]*

[†]Department of Chemistry, Radiochemistry, University of Helsinki, Finland

[§]Department of Biochemistry and Developmental Biology, Faculty of Medicine, University of Helsinki, Finland

[¤]Present address: Turku PET Centre, Department of Chemistry, University of Turku, Finland

*Corresponding author: Anu J. Airaksinen

Department of Chemistry, Radiochemistry, P.O. Box 55, FI-00014 University of Helsinki, Finland. E-mail: anu.airaksinen@helsinki.fi. Present address: Turku PET Centre, University of Turku, Kiinamyllynkatu 4-8, FI-20520 Turku

Table of contents

Figure S1 ^{1}H-NMR of compound 3 .... **1**
Figure S2 ^{13}C-NMR of compound 3 .... **2**
Figure S3 ^{1}H-NMR of SiFA-Tz (6) **3**
Figure S4 ^{19}F-NMR of SiFA-Tz (6) **4**
Figure S5 ^{13}C-NMR of SiFA-Tz (6) **5**
Figure S6 HPLC of [^{18}F]SiFA ([^{18}F]5) **6**
Figure S7 HPLC of oxime formation (two-step method) for [^{18}F]SiFA-Tz ([^{18}F]6) (crude).......... **7**
Figure S8 HPLC (radio- and PDA -detectors) of formulated [^{18}F]SiFA-Tz ([^{18}F]6) **8**
Figure S9 Autoradiography TLC-profile of purified [^{18}F]SiFA-Tz ([^{18}F]6).......... **9**
Figure S10 HPLC of [^{18}F]SiFA-Tz ([^{18}F]6) stability in 1xPBS (90 min) **10**
Figure S11 HPLC of purified [^{18}F]fluoroalbumin ([^{18}F]10).......... **11**
Figure S12 Autoradiography TLC-profile of purified [^{18}F]fluoroalbumin ([^{18}F]10) **11**
Figure S13 Biodistribution of [^{18}F]SiFA-Tz ([^{18}F]6).......... **12**
Figure S14 Biodistribution of [^{18}F]fluoroalbumin ([^{18}F]10) **13**
Figure S15 Radio-TLC profile of [^{18}F]SiFA-Tz ([^{18}F]6) in human plasma stability studies **14**
Figure S16 Radio-HPLC profile of [^{18}F]SiFA-Tz ([^{18}F]6) in human plasma stability studies **14**
Figure S17 Radio-HPLC profile of [^{18}F]SiFA-Tz ([^{18}F]6) metabolites found in blood **15**

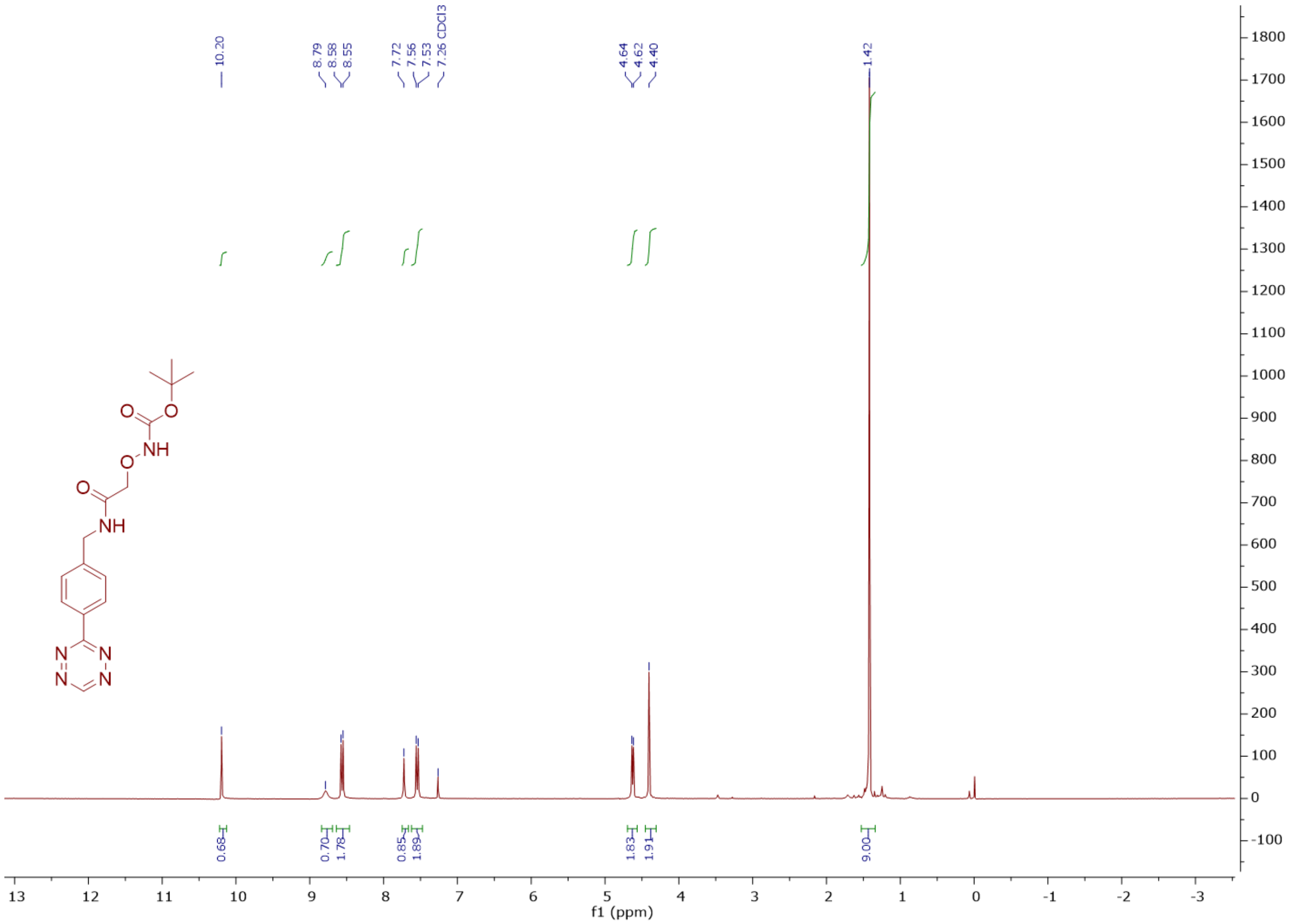

Figure S1. ^{1}H-NMR (300 MHz, $CDCl_3$) of compound **3**.

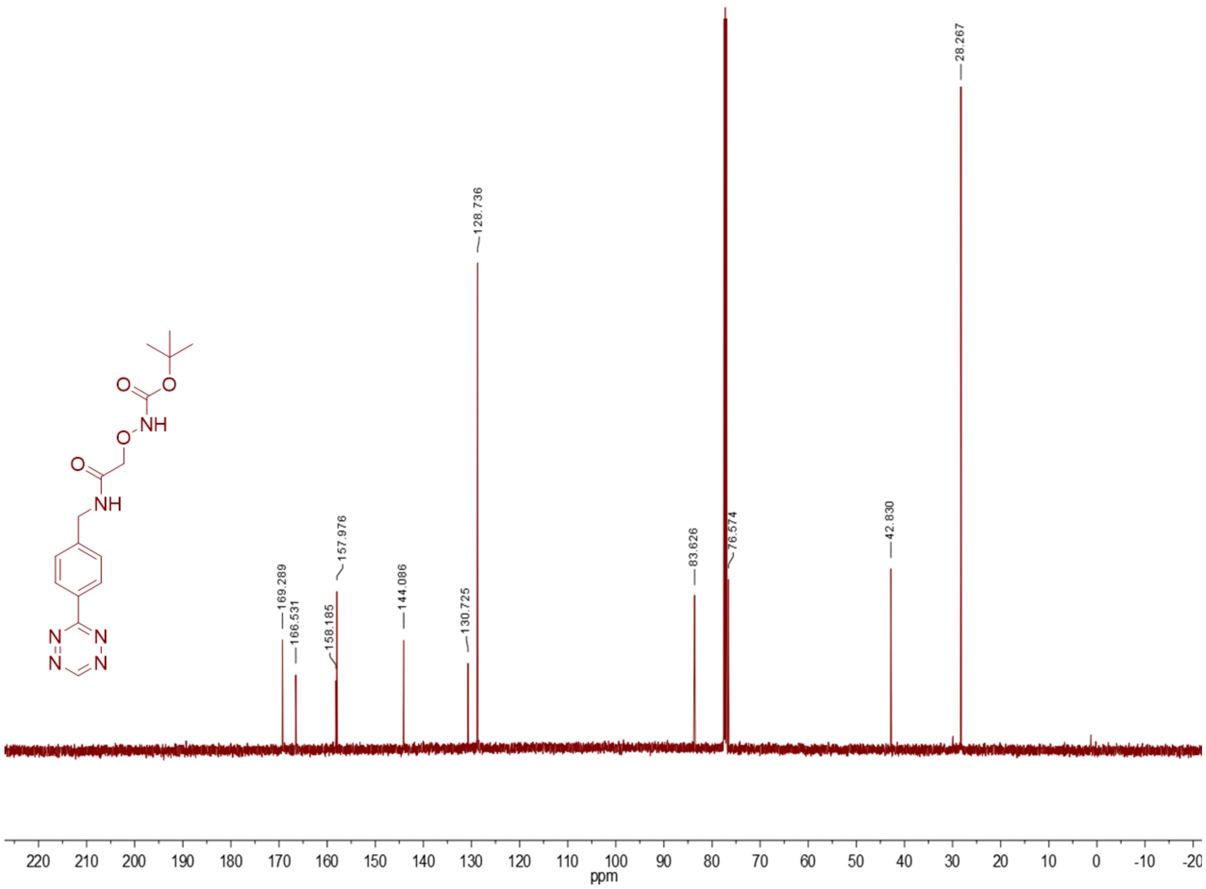

Figure S2. ^{13}C-NMR (75 MHz, $CDCl_3$) of compound **3**.

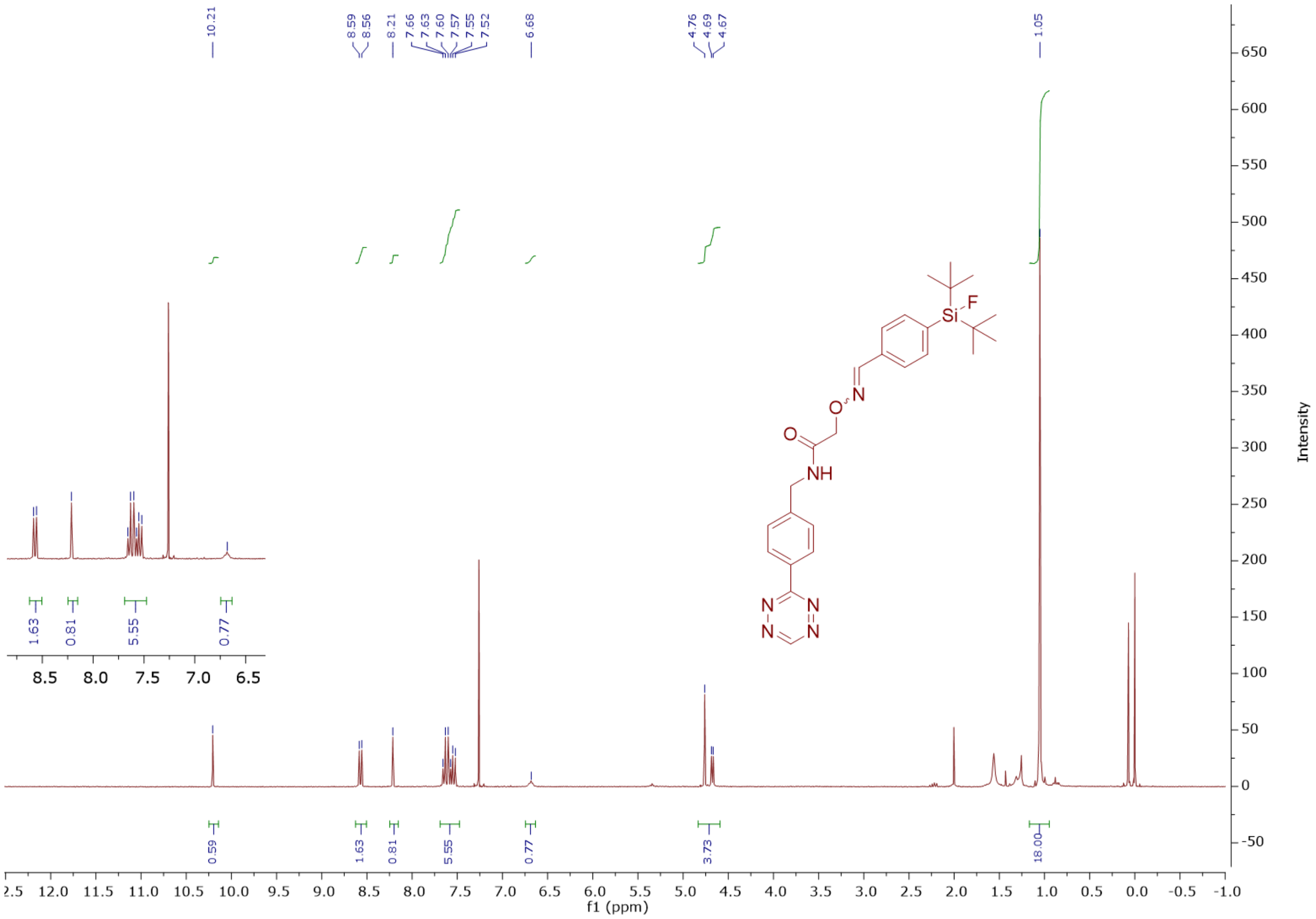

Figure S3. ^{1}H-NMR (500 MHz, $CDCl_3$) of SiFA-Tz (**6**).

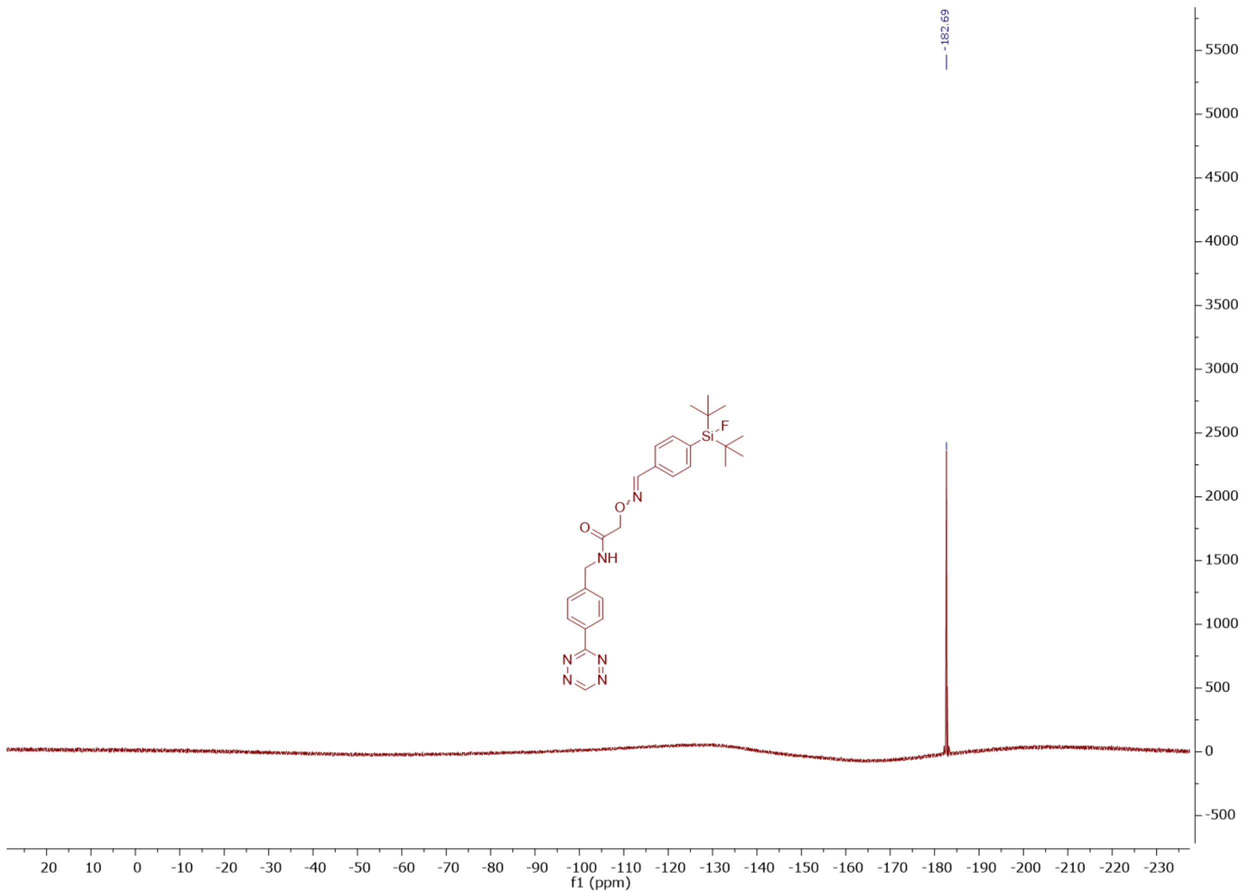

Figure S4. ^{19}F-NMR (282 MHz, $CDCl_3$) of SiFA-Tz (**6**).

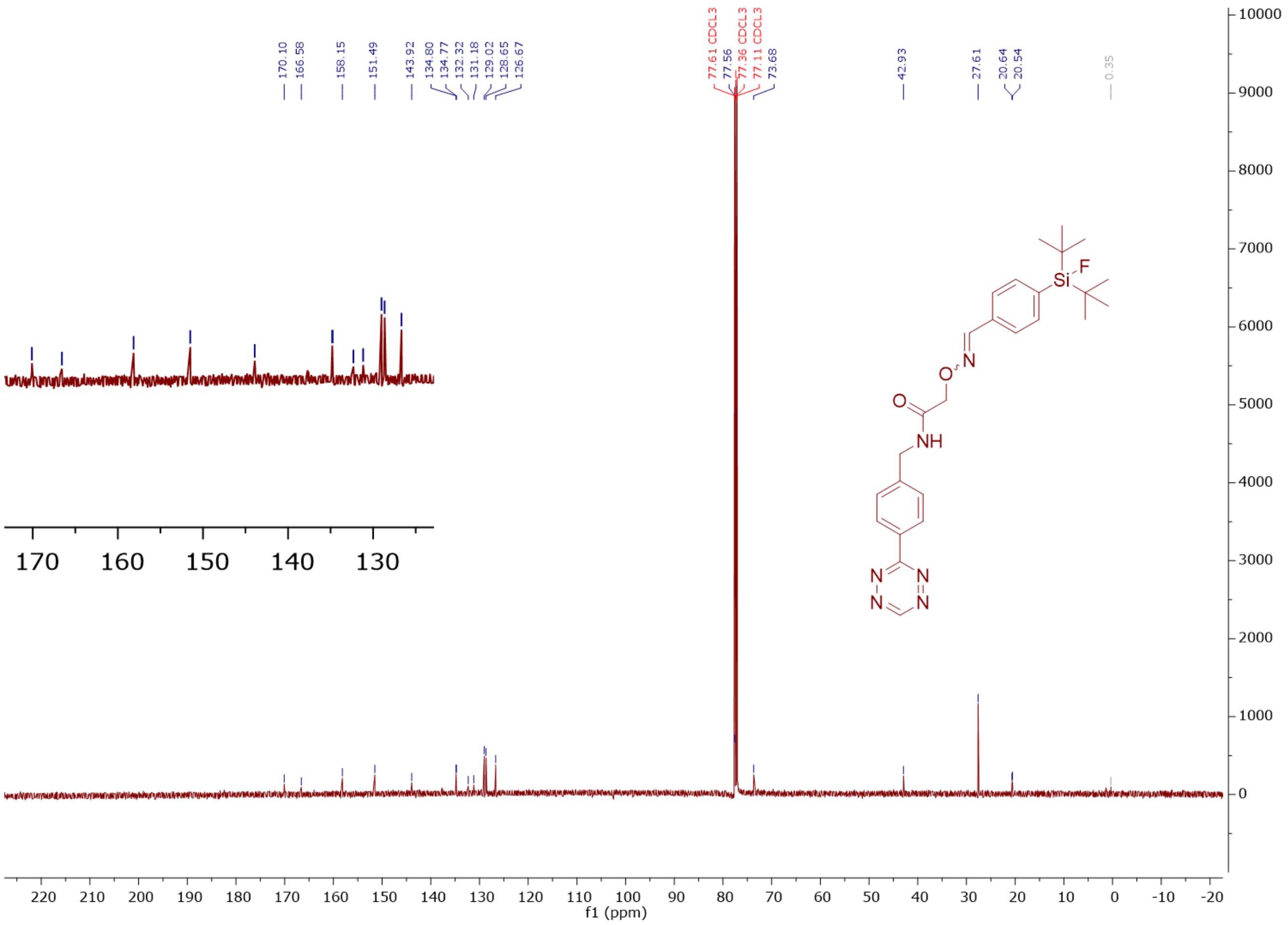

Figure S5. ^{13}C-NMR (126 MHz, $CDCl_3$) of SiFA-Tz (**6**).

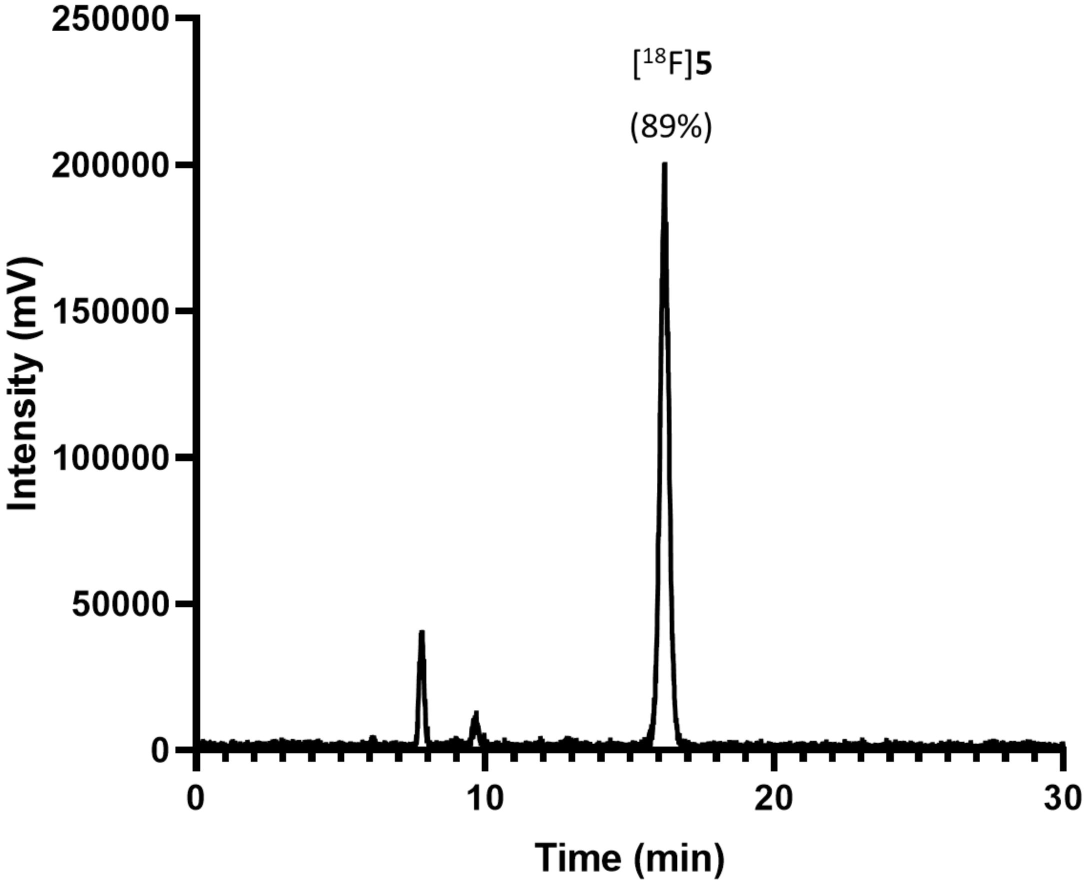

Figure S6. Radio-HPLC chromatogram of crude [^{18}F]SiFA ([^{18}F]**5**) (Radiodetector).

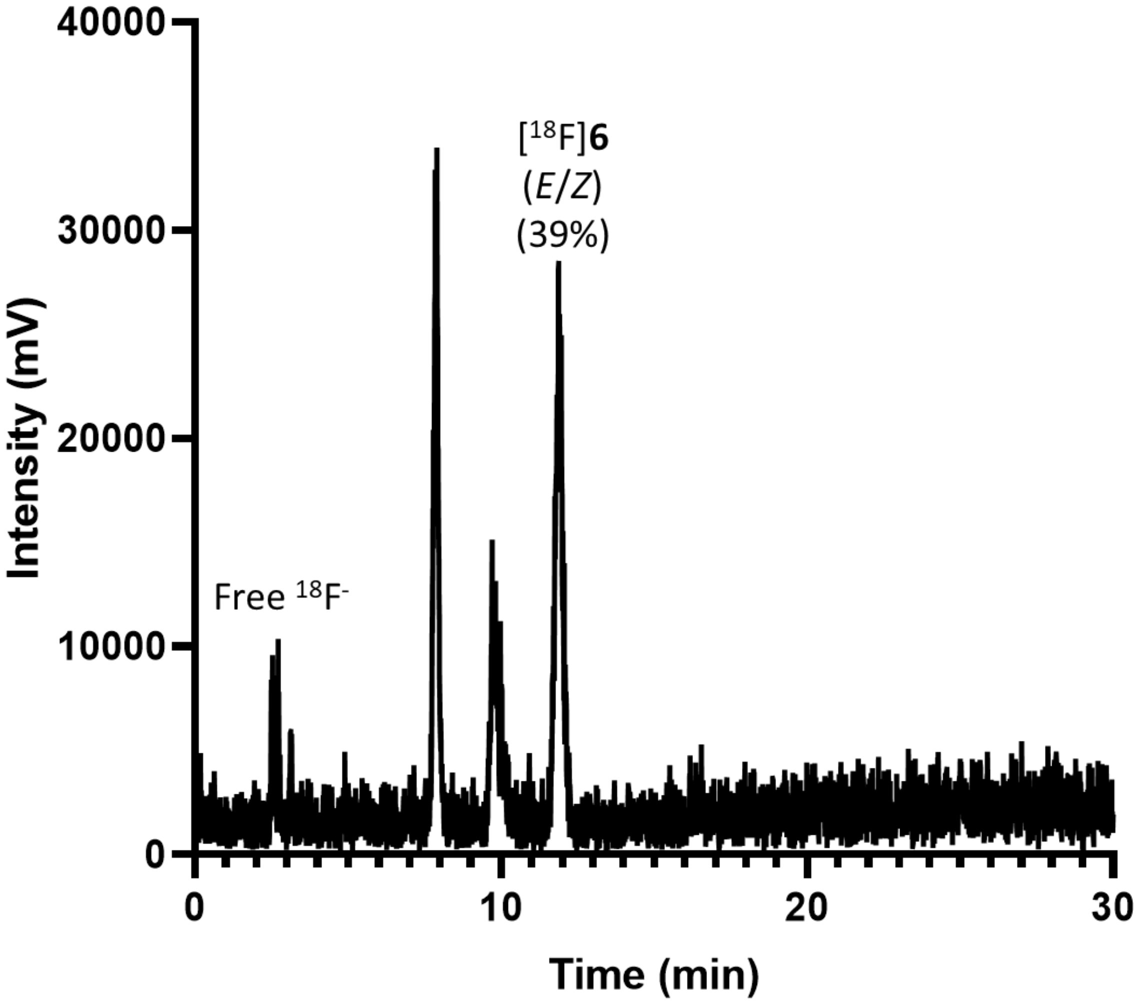

Figure S7. Radio-HPLC chromatogram of oxime formation (two-step method) for [^{18}F]SiFA-Tz ([^{18}F]**6**) crude (radiodetector).

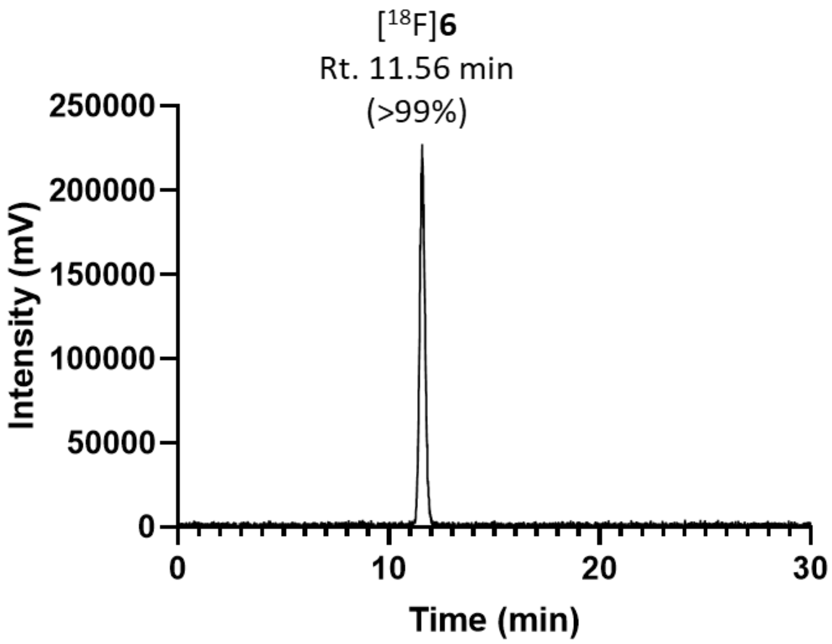

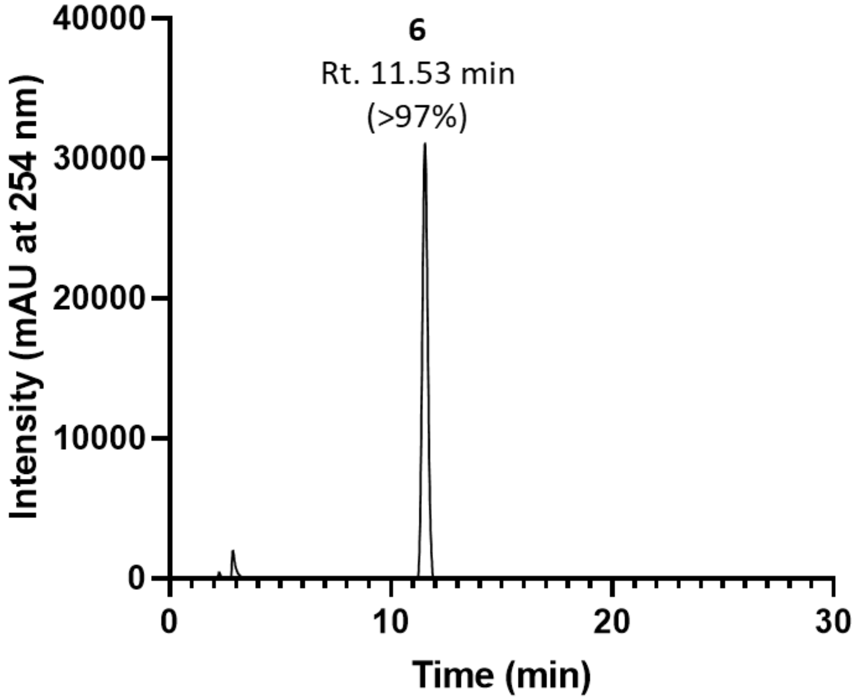

Figure S8. HPLC chromatograms of formulated [^{18}F]SiFA-Tz ([^{18}F]**6**) analyzed by radiodetector (top) and PDA -detector (bottom).

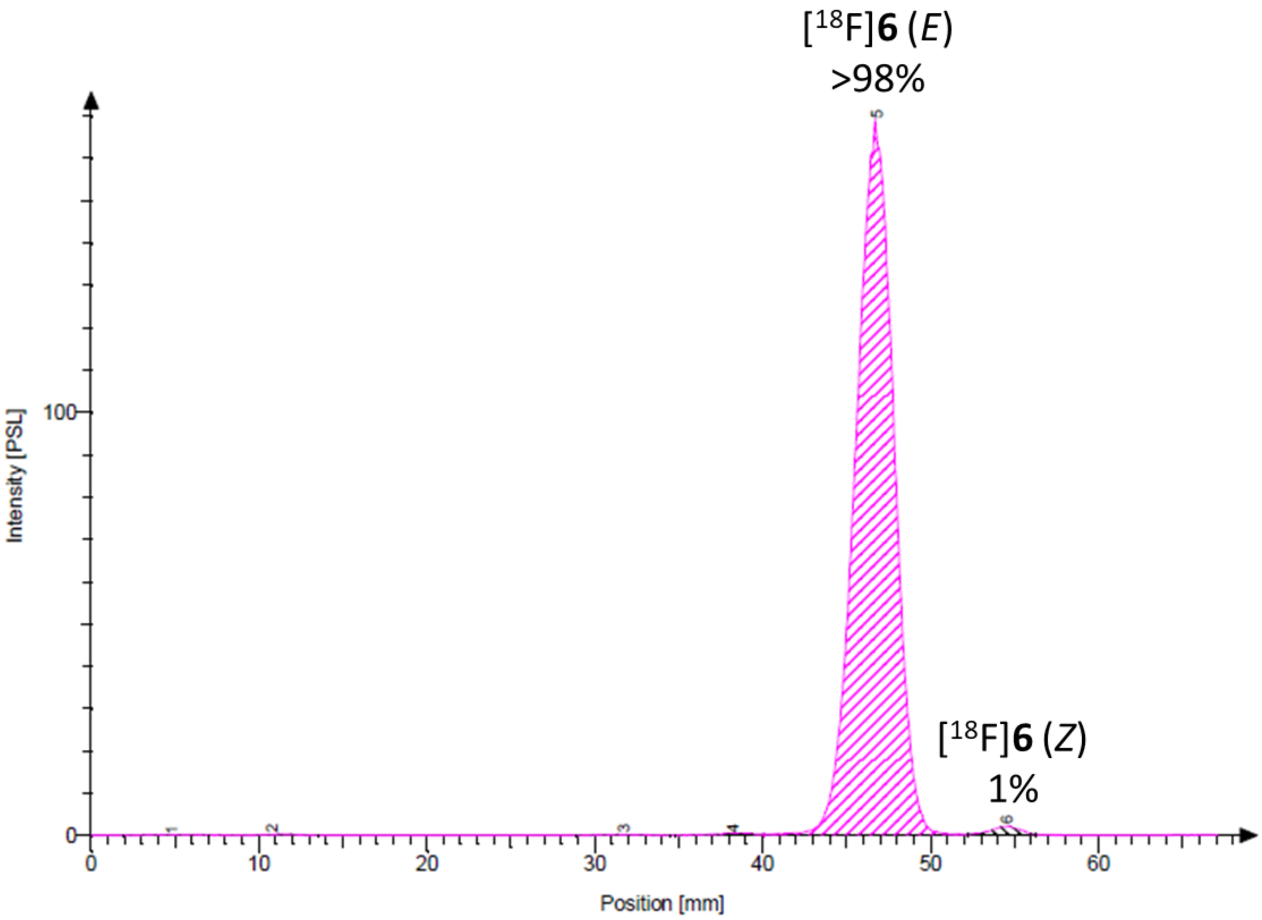

Figure S9. Radio-TLC chromatogram (digital autoradiography) of [^{18}F]SiFA-Tz ([^{18}F]**6**).

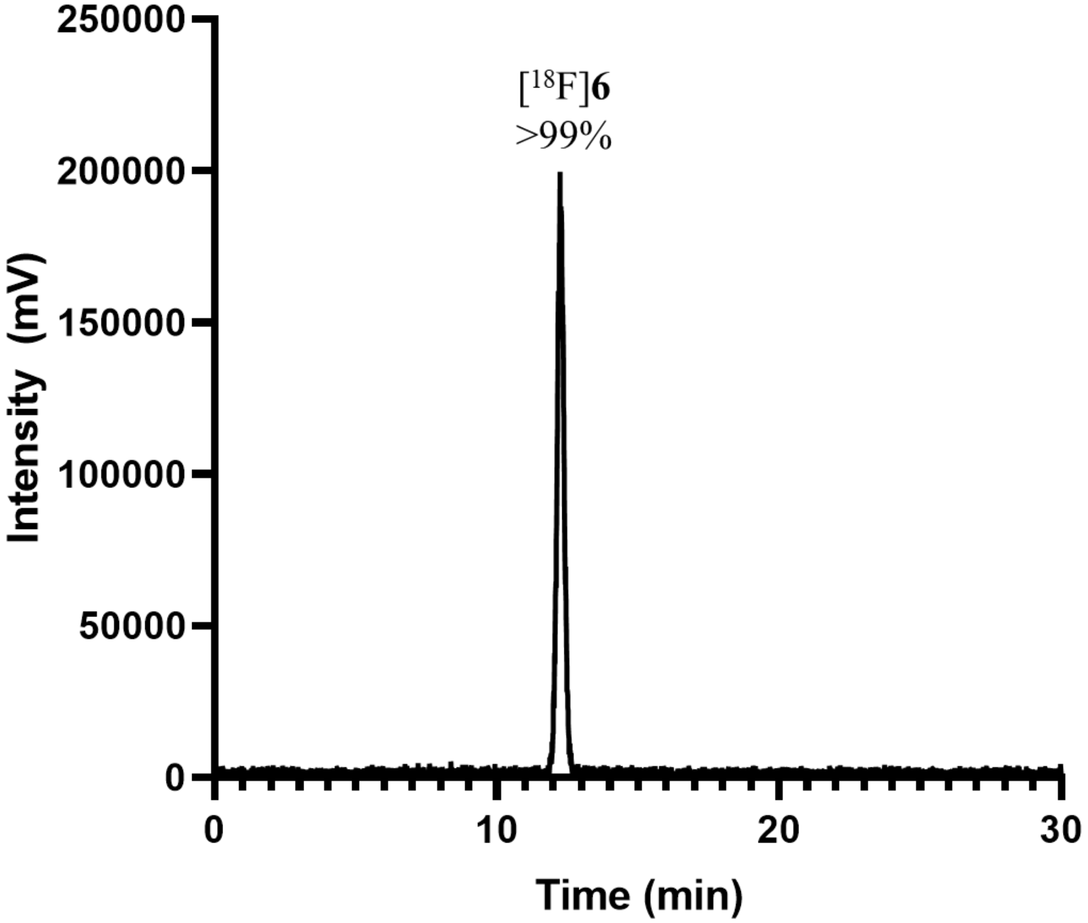

Figure S10. Radio-HPLC chromatogram of [^{18}F]SiFA-Tz ([^{18}F]**6**) stability at 90 minutes in 1×PBS (radiodetector).

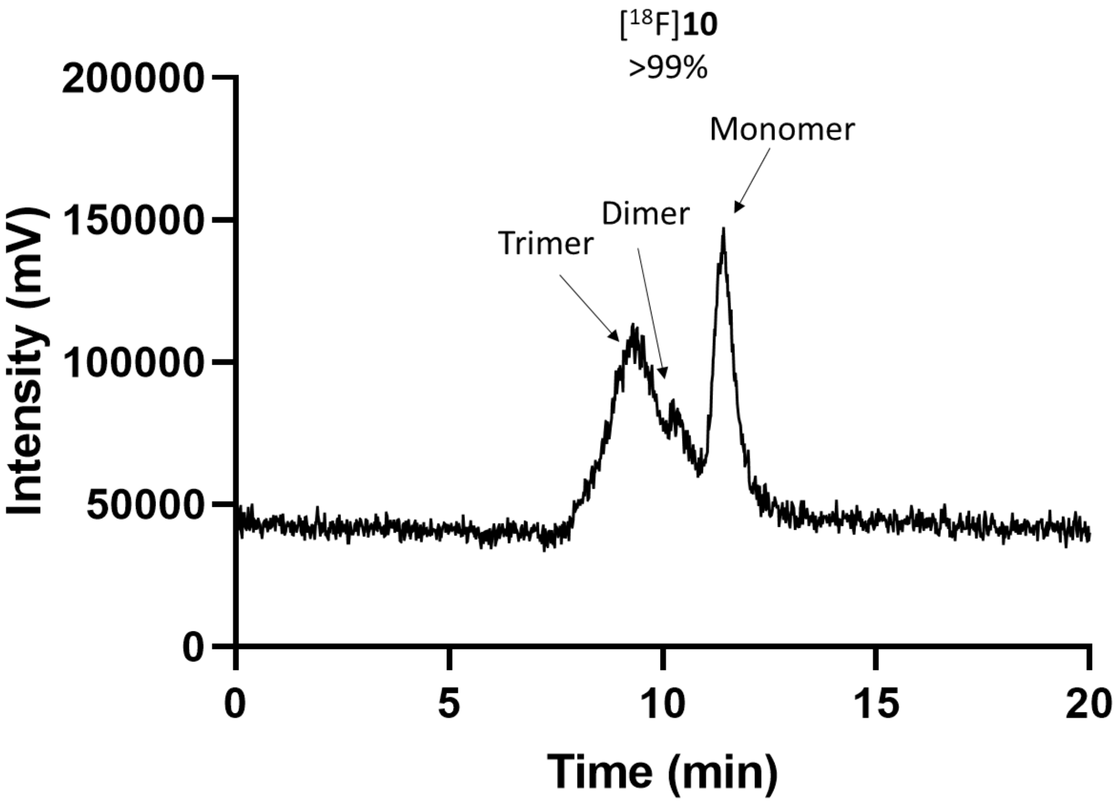

Figure S11. Radio-HPLC chromatogram of [^{18}F]fluoroalbumin ([^{18}F]**10**) (radiodetector).

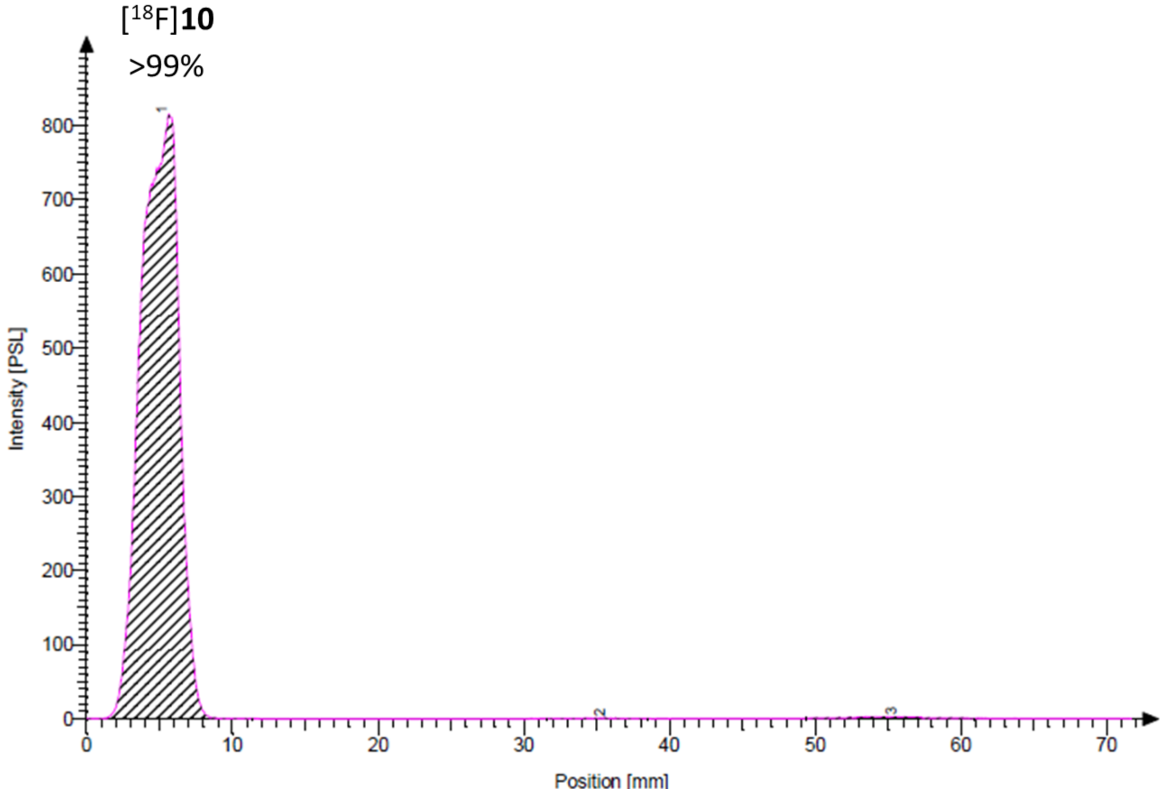

Figure S12. Radio-TLC chromatogram (digital autoradiography) of [^{18}F]fluoroalbumin ([^{18}F]**10**).

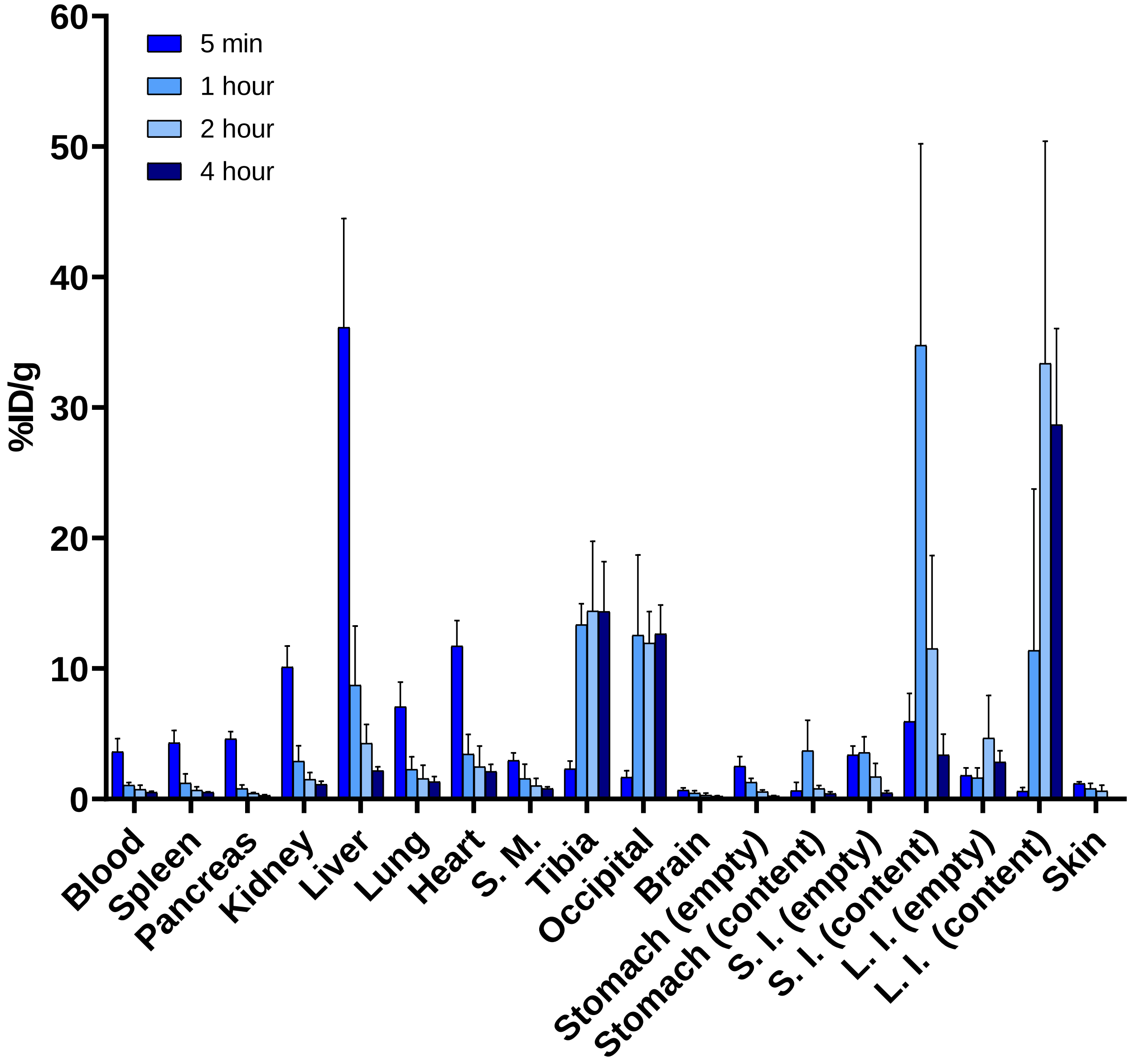

Figure S13. Biodistribution of radioactivity after intravenous administration of [^{18}F]SiFA-Tz ([^{18}F]**6**) demonstrating fast clearance from circulation, hepatobiliary excretion and high bone uptake at 60 minutes post-injection.

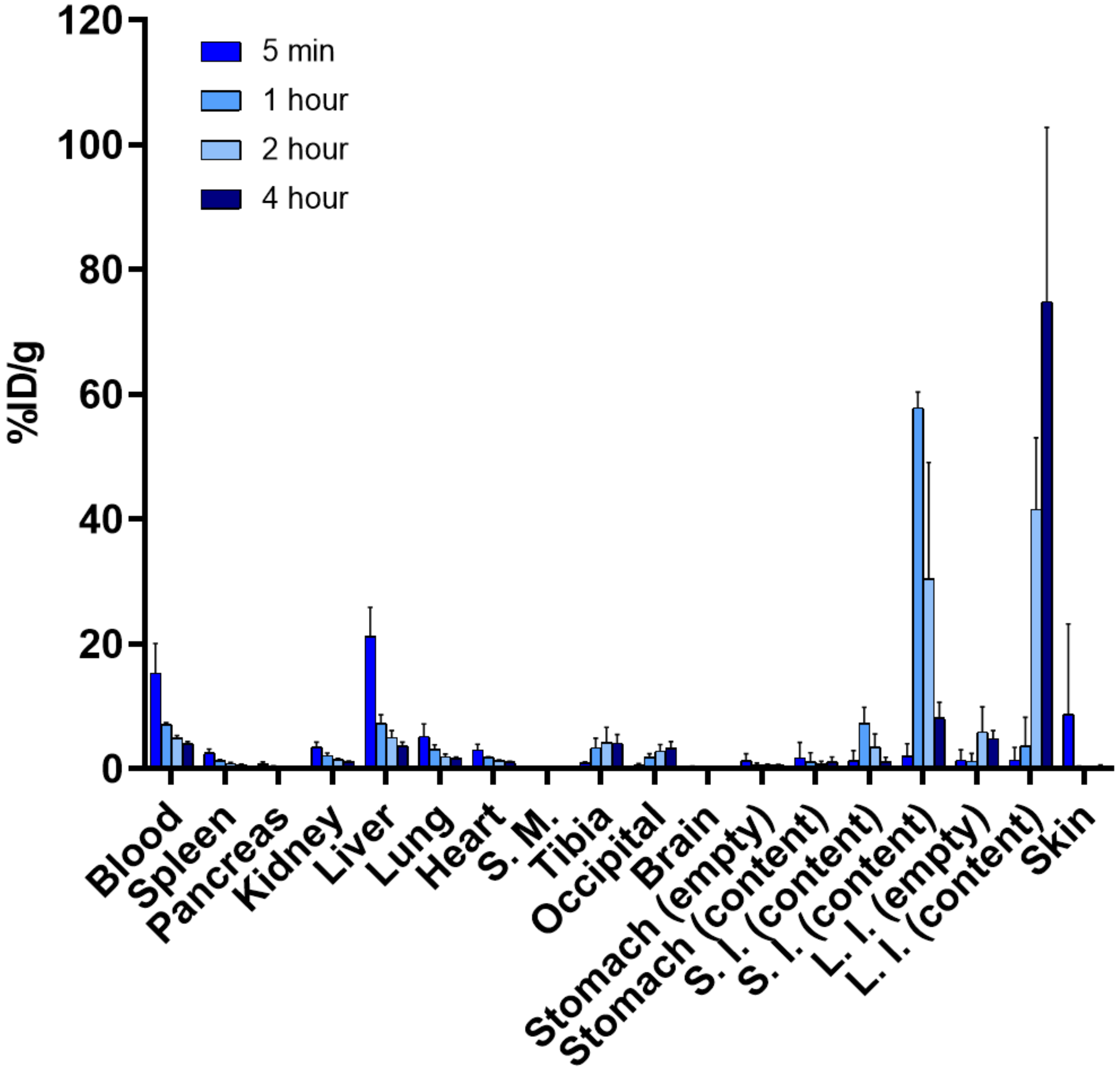

Figure S14. Biodistribution of radioactivity after intravenous administration of [^{18}F]fluoroalbumin ([^{18}F]**10**) demonstrating prolonged residence time in circulation.

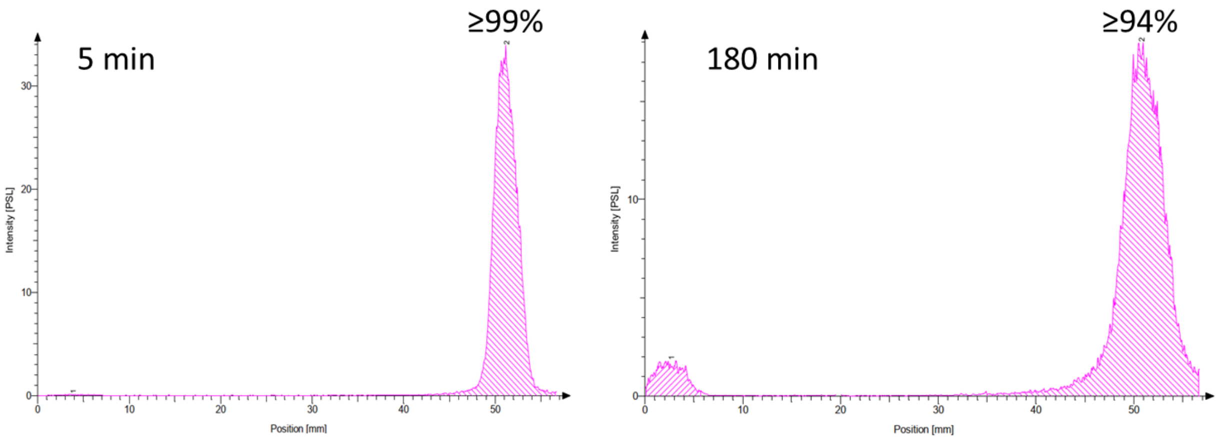

Figure S15. Radio-TLC (digital autoradiography) chromatograms of [^{18}F]SiFA-Tz ([^{18}F]**6**) in human plasma stability studies show minor defluorination of the radiotracer at 180 minutes after start of incubation.

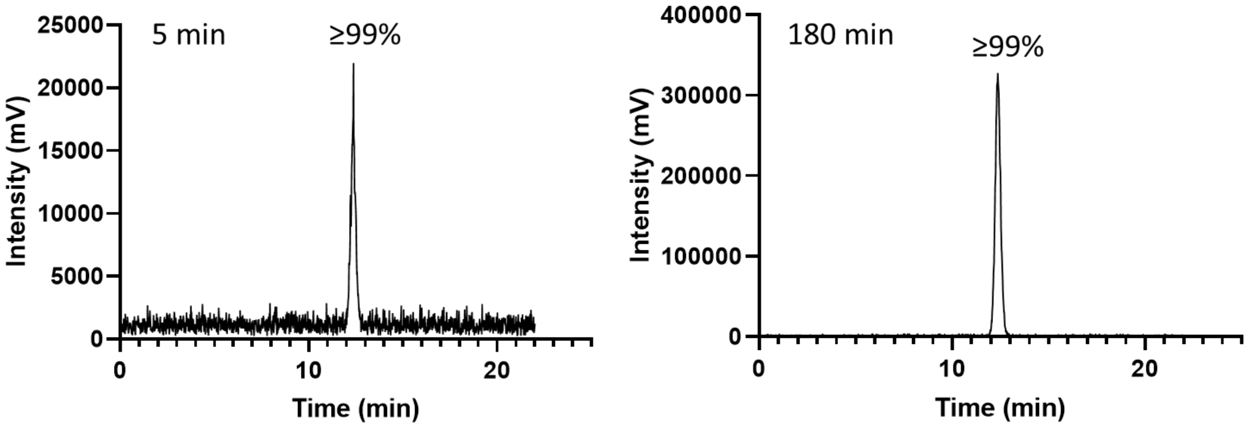

Figure S16. Radio-HPLC chromatograms of [^{18}F]SiFA-Tz ([^{18}F]**6**) in human plasma stability studies demonstrating no detectable decomposition.

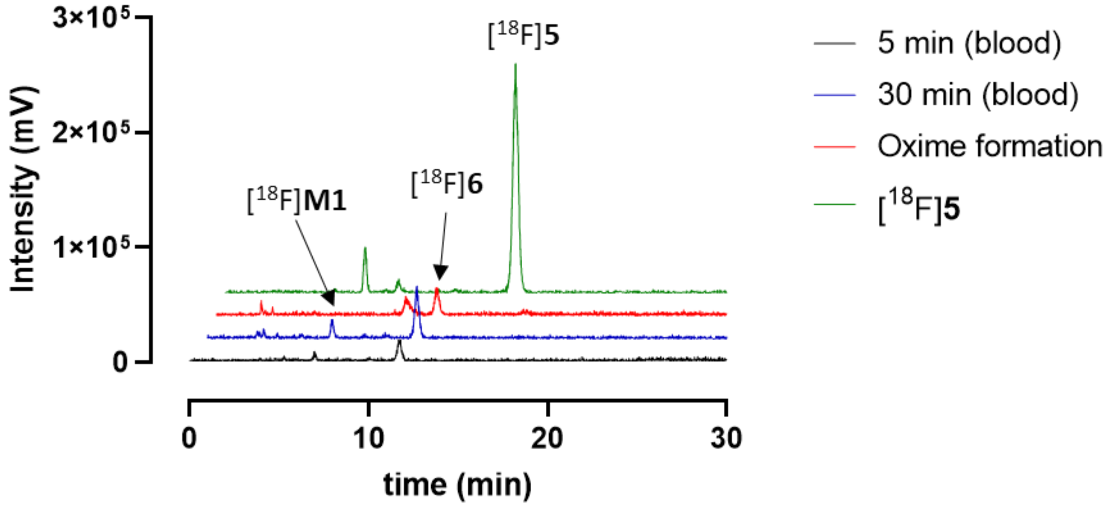

Figure S17. Radio-HPLC chromatograms of radiolabeling of [^{18}F]SiFA-aldehyde ([^{18}F]**5**), oxime formation producing [^{18}F]SiFA-Tz ([^{18}F]**6**) and metabolites (*t* = 5 min, 30 min) found in mouse blood after intravenous administration of [^{18}F]SiFA-Tz ([^{18}F]**6**).
